# Natural medicines-derived carbon dots as novel oral antioxidant administration strategy for ulcerative colitis therapy

**DOI:** 10.1186/s12951-024-02702-2

**Published:** 2024-08-27

**Authors:** Tong Wu, Xue Bai, Yue Zhang, Ertong Dai, Jinyu Ma, Cai Yu, Chenxin He, Qiannan Li, Yingxin Yang, Hui Kong, Huihua Qu, Yan Zhao

**Affiliations:** 1 School of Chinese Materia Medica, Beijing University of Chinese Medicine, Beijing, 100029 China; 2 School of Traditional Chinese Medicine, Beijing University of Chinese Medicine, Beijing, 100029 China; 3 School of Life Science, Beijing University of Chinese Medicine, Beijing, 100029 China; 4 Qingdao Eighth People’s Hospital, Qingdao, 266100 China; 5 Department of Endocrine, Beijing Daxing District Hospital of Integrated Chinese and Western Medicine, Beijing, 100163 China; 6 Department of Traditional Chinese Medicine, Beijing Daxing District Hospital of Integrated Chinese and Western Medicine, Beijing, 100163 China; 7 Centre of Scientific Experiment, Beijing University of Chinese Medicine, Beijing, 100029 China

**Keywords:** Carbon dots, Natural medicine, Antioxidant, Ulcerative colitis, Intestinal flora

## Abstract

**Background:**

Ulcerative colitis (UC) is a chronic intestinal inflammation, resulting in a global healthcare challenge with no real specific medicine. Natural medicines are recognized as a potential clinical alternative therapy, but their applications are limited by poor solubility and low bioavailability.

**Results:**

In this work, inspired by the natural medicines of ancient China, novel functional carbon dots derived from Magnetite and Medicated Leaven (MML-CDs) were synthesized by hydrothermal method, and confirmed their ultrasmall nano-size (3.2 ± 0.6 nm) and Fe doped surface structure, thereby with excellent gastrointestinal stability, remarkable capabilities in eliminating ROS, and highly biocompatibility. With no external stimuli, the oral administration of MML-CDs demonstrated obvious alleviation to UC. Further experiments pointed that MML-CDs could improve hemostasis capability, suppress inflammation reactions and oxidative stress, and up-regulate the expression of tight junction proteins. Furthermore, MML-CDs also showed well regulation in the dysbiosis of intestinal flora.

**Conclusion:**

Overall, above evidence reveals that green-synthesized MML-CDs can significantly alleviate intestinal bleeding, inhibit colon inflammation, and repair colonic barrier damage, further regulating intestinal flora and intestinal inflammation microenvironment. Our findings provide an efficient oral administration of MML-CDs as a novel therapy strategy for ulcerative colitis.

**Graphical Abstract:**

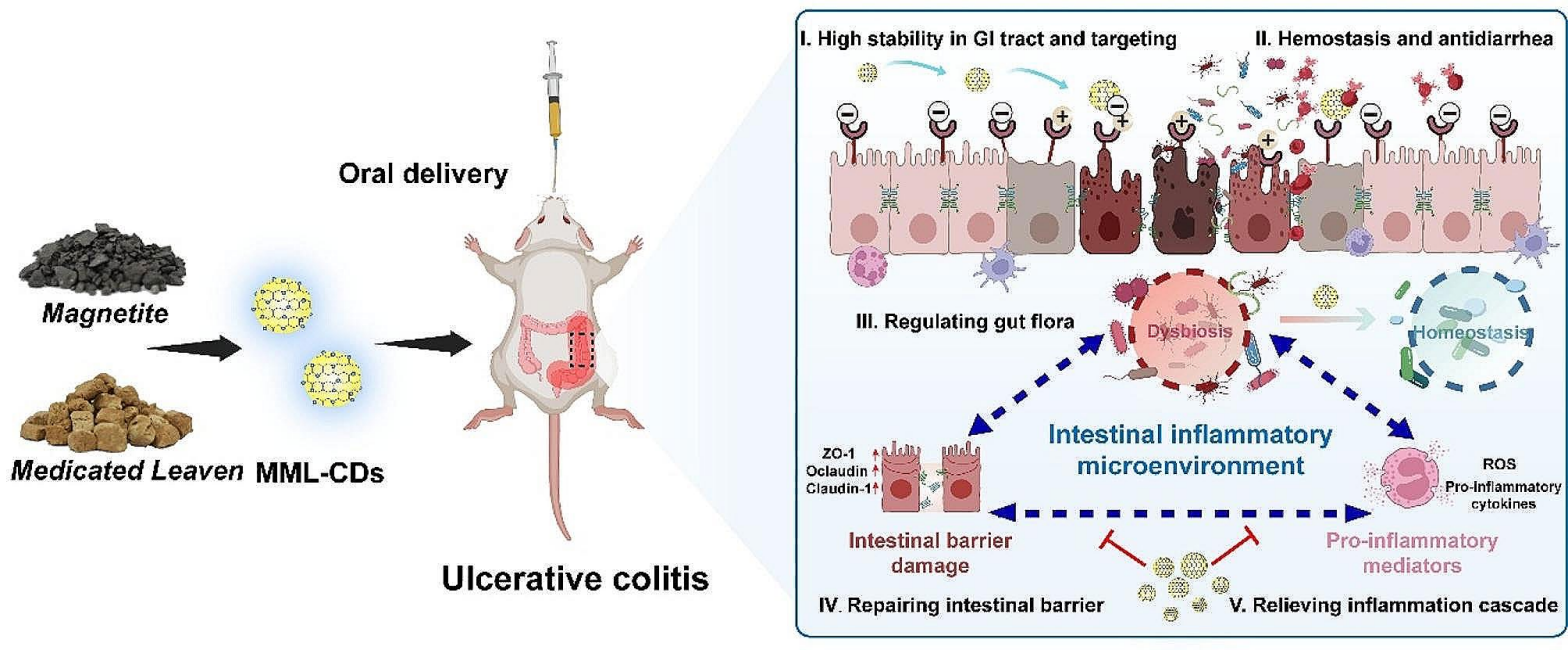

**Supplementary Information:**

The online version contains supplementary material available at 10.1186/s12951-024-02702-2.

## Introduction

Ulcerative colitis (UC), recognized as a chronic and multifaceted inflammatory bowel disorder (IBD), has become a global healthcare challenge due to a continuous increase in incidence in recent years [1]. UC is generally characterized by colonic inflammation and the distressing symptomatology of bloody diarrhea [2], which initially manifests in the rectum and may extend to more proximal segments of the colon. The development of UC is intricately linked to ongoing episodes of diffuse mucosal inflammation, compromised intestinal epithelial barrier, and imbalance in the colonic microbiota [3, 4]. The first-line therapeutic agents for UC typically include biologics, steroids, and immunosuppressants [5]. However, when these treatments are administered orally, they often demonstrate low bioavailability and may present potential biotoxicity to patients with UC. [6, 7] Hence, it is urgent to investigate innovative oral administration strategies for UC therapy for potential clinical application.

A growing number of studies have confirmed that the intestinal mucosa afflicted by UC is subjected by overproduced reactive oxygen species (ROS) in comparation with the health colon tissue [8]. The overproduction of ROS and continuous oxidation stress not only inflict direct damage to or even the destruction of the intestinal microvilli’s integrity, but also perpetuate the vicious cycle between ROS and inflammation, culminating in pathological inflammatory responses [9–11]. Moreover, excess amount of ROS acts as the terminal electron receptors for the anaerobic respiration of facultative anaerobic bacteria, ultimately promoting the proliferation of harmful bacteria and contributing to intestinal dysbiosis [12]. With the development of nanomedicine, numerous of experimental evidence revealed that many nano-size bioactive materials have benefit in alleviation of inflammation and oxidative stress, including metal-based nanozyme [13], hydrogel [14], micelle [15] and nanoparticles [16, 17]. Although these materials basically demonstrate effective scavenging capacity of ROS, some studies had also indicated that the ROS scavenging ability of these materials may be affected or reduced due to the complex acid-base digestive tract and protease [18, 19]. Furthermore, UC is a clinical chronic disease persisting for diseases, and the long-term presence of metal-based nanoparticles inevitably leads to the release of metal ions to absorb by the digestive tract, potentially resulting in biotoxicity [20].

In recent years, carbon dots (CDs), known as a zero-dimensional nanomaterial, have gained increasing attention for their potential applications in biotherapy, owing to their ultrasmall size (< 10 nm), stable surface charge, functional chemical groups, and high biocompatibility [21–23]. Recent exploration has confirmed a number of CDs have the excellent potential to serve as nanomedicine in digestive disease [24, 25]. Among them, based on green synthesis process, low cost and ROS scavenging ability, natural medicine-derived CDs (NM-CDs) naturally attracted considerable attention from clinical scientists [22], which can be an efficient strategy to improve low solubility and poorly bioavailability of natural medicine. The carbon dots derived from *Atractylodes macrocephala* (CAM-CDs) not only alleviated inflammatory and oxidant stress by regulating the NF-κB/NLRP3 axis, but also regulated the microbial diversity and species composition of gut microbiota to improve gastric ulcer [26]. The broccoli water extract-derived CDs (BWE-CDs) had excellent ROS scavenging ability to DPPH· (2,2-diphenyl-1-picrylhy-drazyl) and ABTS+· (2,2’-Azinobis-(3-ethylbenzthiazoline-6-sulphonate)), and their anti-UC mechanism was related with the direct reaction between CDs and ROS [27]. These therapy activities may be related with surface functional groups of NM-CDs preserving from their natural medicine precursor [28]. In addition, recent study demonstrated that numerous NM-CDs can accelerate hemostasis through activation of platelets and coagulation pathways [29]. In the investigation of *Rhei radix rhizoma*-based carbon dots (RRR-CDs) [30], RRR-CDs alleviated colonic inflammation, upregulated the expression of tight junction proteins, and inhibited bleeding from ulcerated surfaces to reduce intestinal infections. Although these explorations provide valuable insights and data into the UC treatment potential of NM-CDs, there are limited concerns regarding the gastrointestinal stability of NM-CDs, changes in antioxidant activity, further therapeutic mechanisms, and scant evidence exploring the potential bioactivity of NM-CDs derived from dual precursors instead of a single one [31].

**Scheme 1:**
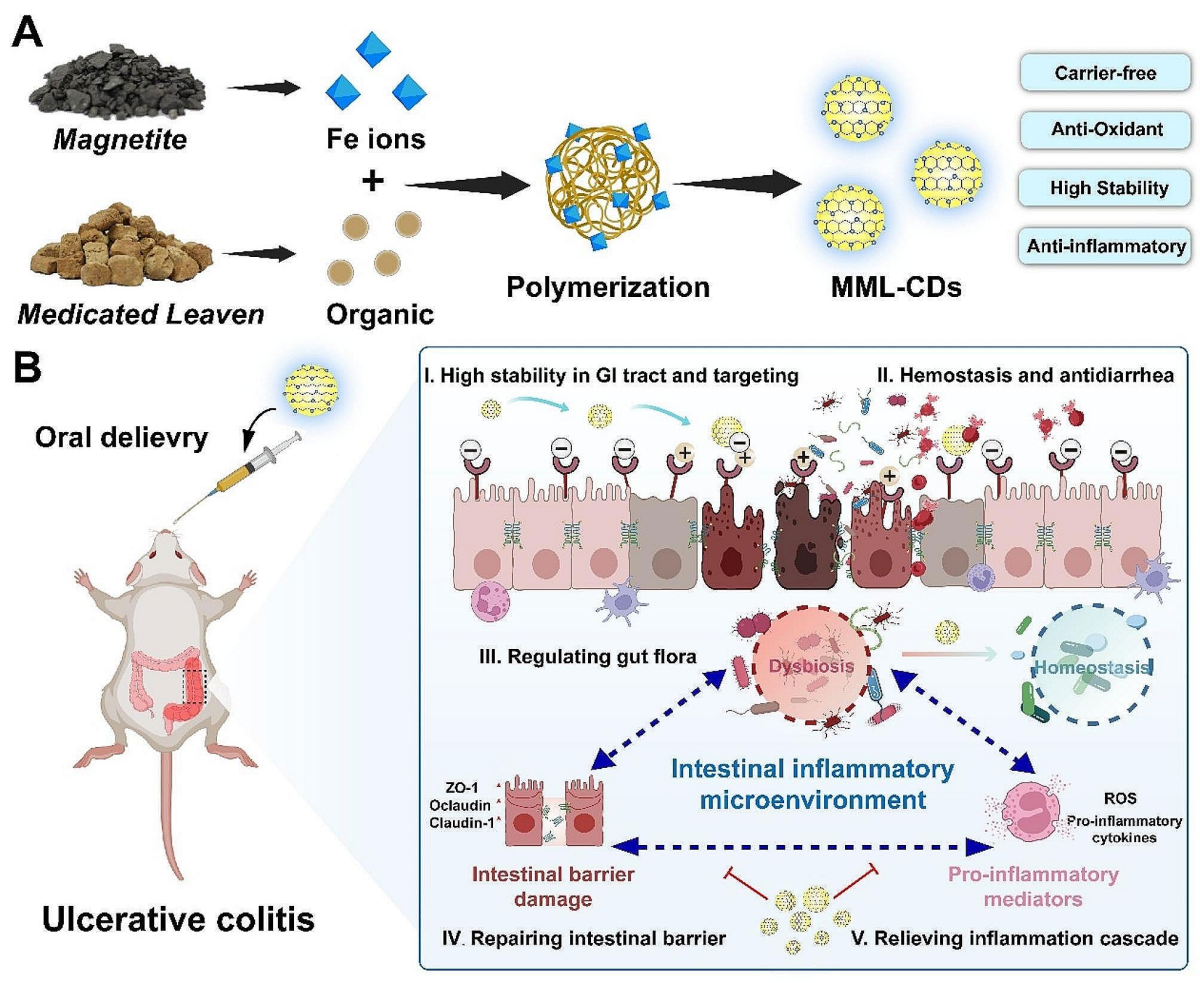
Schematic illustration of (**A**) the synthesis of MML-CDs and (**B**) the oral administration of MML-CDs to improve colonic microenvironment for ulcerative colitis therapy in mice

The synergistic effect of Magnetite (Ci-shi) and Medicated Leaven (Shen-qu) from Traditional Chinese Medicine (TCM) have been applied in clinical therapy for ulcerative colitis since the Tang Dynasty of Ancient China. However, few studies have focused their material basis and further mechanism. Based on this inspiration, we firstly introduced that green carbon dots synthesized from Magnetite and Medicated Leaven (MML-CDs) using one-step hydrothermal method without any chemical agents. MML-CDs demonstrated a Fe-doping surface structure, excellent gastrointestinal stability, highly biocompatibility, and remarkable capabilities in eliminating ROS and alleviating oxidative stress in Caco-2 cells. Furthermore, MML-CDs exhibited promising therapeutic effects on dextran sodium sulfate (DSS)-induced ulcerative colitis in mice. Subsequent experiments indicated that MML-CDs could improve UC by accelerating hemostasis, regulating inflammation reactions and oxidative stress, and repairing colonic barrier damage (Scheme 1). The potential treatment mechanism of MML-CDs was further studied using 16 S rDNA sequencing. Thus, this work may offer valuable insights for the further treatment strategies of MML-CDs for UC and other inflammation diseases.

## Results and discussion

### Synthesis and characterization of MML-CDs

**Fig. 1:**
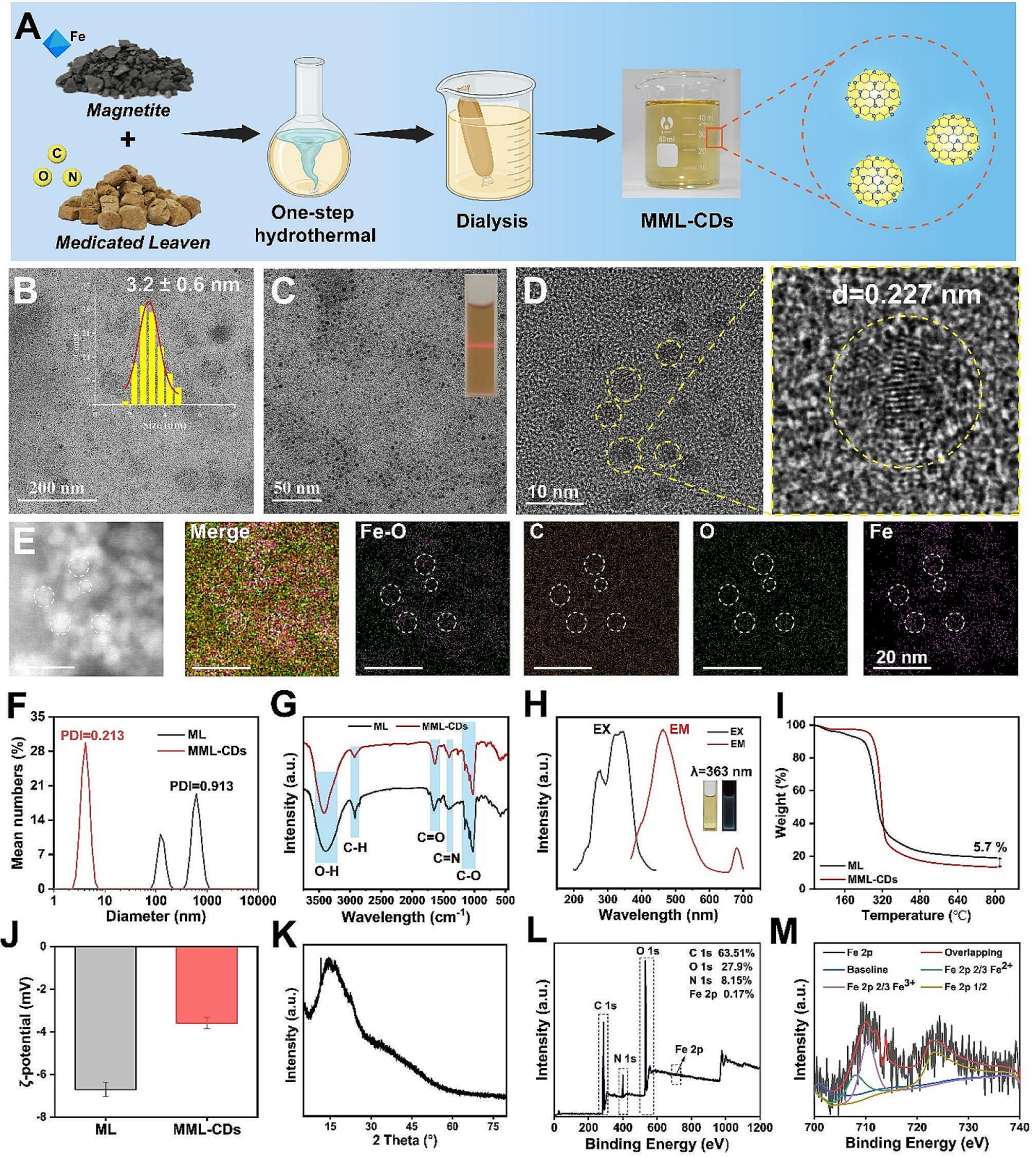
Synthesis and characterization of MML-CDs. (**A**) Schematic illustration of MML-CDs. (**B**-**C**) TEM images of MML-CDs, scale bars: 200 nm and 50 nm, respectively. (**D**) HR-TEM images of MML-CDs, scale bars, 10 nm. (**E**) TEM image and elemental mapping of MML-CDs, scale bars: 20 nm. (**F**) DLS measurements of ML and MML-CDs. (**G**) FT-IR spectra of ML and MML-CDs. (**H**) Fluorescence excitation (black) and emission (red) spectra of MML-CDs. (**I**) TGA cruces of ML and MML-CDs. (**J**) ζ-potentials of ML and MML-CDs (*n* = 3). (**K**) XRD pattern of MML-CDs. (**L**) Full survey spectrum XPS spectrum of MML-CDs. (**M**) The high-resolution of Fe 2p XPS spectrum of MML-CDs

MML-CDs were synthesized using one-step hydrothermal method (Fig. 1A), and the preparation process was inspired by the processing method in TCM (Fig. S1). The resulting MML-CDs powder had a yellow-brown appearance with good water-solubility, and was diluted with deionized water (DW) for characterization.

The obtained MML-CDs exhibited a uniform spheroid-like nanostructure ranging from 2 to 5 nm in size, with an average particle diameter of 3.2 ± 0.6 nm (Fig. 1B, C), which indicated an excellent biological barrier penetration of MML-CDs [32, 33]. Compared with the TEM image of the single ML solution (Fig. S2), MML-CDs displayed a clearer and simpler nanostructure without precursor residues and salt crystallization. The lattice space of carbon core on MML-CDs exhibited a graphite-like cryptal lattice (100) distance of 0.227 nm by HR-TEM [34]. (Fig. 1D) These results are similar to the previous studies on natural medicine-derived CDs [22]. Similarly, CDs synthesized from *Carthamus tinctorius L.* or *Angelica sinensis* with nano-size less than 5 nm could uptake by Hela cells and alleviate joint capsule inflammation and articular cartilage destruction [35]. The CDs from *Fuligo Plantae* with a particle size distribution between 1.4 and 3.2 nm exhibited well penetration ability to gastric mucosal barrier after administration orally to improve tissue damage and inflammation, additionally maintaining well stability in water and NaCl solution [36]. Moreover, carbonized *Pollen Typhae*-derived CDs showed the ability to penetrate the glomerular filtration barrier with the ultrasmall size less than 5 nm [33], accordingly improving acute kidney injury. Thereby, these results offer enough evidence to potential biological barrier penetration of natural medicine-derived CDs. Next, DLS test observed that MML-CDs solution have significant Tyndall effect (the Inset image of Fig. 1C), and demonstrated a narrower distribution (hydrodynamic diameter: 4.1 nm; PDI: 0.213) compared with single ML solution (hydrodynamic diameter:123.7 and 621.5 nm; PDI: 0.913), suggesting the stable hydrodynamic size of MML-CDs (Fig. 1F). Thus, the above characteristics confirmed that MML-CDs with ultrasmall nano-size and high purity were efficiently synthesized.

The optical properties of MML-CDs were further assayed. The solution of MML-CDs in UV-vis spectrum has a weak absorption band at about 275 nm (Fig. S3), attributing to the π-π* electron transition of the conjugated C = C bonds and aromatic sp^2^ domains on the carbon core [37]. In fluorescence spectrum analysis, MML-CDs solution showed weak blue fluorescence at 365 nm while it was a yellow aqueous solution at daylight (The Inset image of Fig. 1H). Moreover, MML-CDs detecting by FL spectrum showed the maximum emission wavelength and maximum excitation wavelength at 463 nm (blue-infrared region) and 363 nm, respectively (Fig. 1H). We speculated that the blue emission of MML-CDs originated from the present polyphenol structure and sp^2^ hybrid atomic domains of carbon core [29]. However, the emission peaks of MML-CDs ranging from 300 to 400 nm exhibited excitation-independent manner (Fig. S4), which indicted that MML-CDs may have stable photostability.

Furthermore, to explore the potential structure characteries of MML-CDs, we performed a series of chemical structure tests. Firstly, the individually scatted bright dots in the aberration corrected high-angle annular dark-field scanning TEM images confirmed the atomic dispersion of C, O, N and Fe atoms (Fig. 1E). The information indicated the presence of Fe atom in the surface of MML-CDs, which has been hardly reported in the previously carbon dots derived from natural medicine. FT-IR spectra exhibited the changes of chemical functional groups of MML-CDs (Fig. 1G). Compared to single ML powder, MML-CDs have similar chemical groups distribution of O-H (3390 cm^− 1^), -CH_2_-/-CH_3_ (2912 cm^− 1^), C = O/C = N (1649 cm^− 1^), and C-O (1411 cm^− 1^), suggesting that MML-CDs retain the structure of functional groups of ML such as polyphenols structures [26]. The surface exposed polyphenol structures can interact with the transporters and promoting cellular endocytosis, thereby benefiting to nanoparticles crossing the biological barrier [38]. Meanwhile, a newly peak at 1261 cm^− 1^was found in MML-CDs spectrum, which may be related with the coordination of Fe ions. TGA analysis revealed MML-CDs have higher T_d10_ value, indicating that MML-CDs have high thermal stability (Fig. 1I). Additionally, there is a 5.8% weight difference between MML-CDs and single ML sample, indicating the successful coordination of Fe ion on MML-CDs [39]. The ζ-potentials result of MML-CDs have shown lower value (-3.51 mV) than single ML solution (-6.82 mV), which confirmed that MML-CDs have negative surface potential after coordinating between Fe ion and ML precursor (Fig. 1J). The result of XRD exhibited a distinct diffraction peak with small amounts of sharp peaks (Fig. 1K), which also confirmed amorphous carbon structure coordinated with Fe ion. Furthermore, BET surface investigated the surface area, pore width and pore volume of MML-CDs (Fig. S5). The results showed that the surface area of MML-CDs is 7.518 m^2^/g, while the calculated pore width and pore volume are 3.849 nm and 0.015 cm^3^/g, respectively. These data confirmed that MML-CDs is microporous.

The XPS technology was performed to identify surface chemical groups and elements composition of MML-CDs. As shown in Fig. 1L, the predominant elements percentages of MML-CDs were C, O, N and Fe atoms as 63.51%, 27.9%, 8.15% and 0.17%, respectively. The high-resolution C 1s spectra display the distinct peaks corresponding to C-C/C = C, C-O and C-N/C = O [39], and high-resolution O 1s and N 1s spectra separately correspond to C = O, C-O, C-N and N-H [40] (Fig. S6). Additionally, high-resolution Fe 2p spectrum determined a doublet at 708.7 and 723.1 eV, which are ascribed to the Fe 2p_3/2_ and Fe 2p_1/2_ signals, respectively (Fig. 1M). The Fe 2p_3/2_ peak can be fitted into two peaks with the BEs of 707.9 and 710.5 eV, corresponding to Fe^3+^ and Fe^2+^ with the ratio of the two fitting peak areas close to 2:1. The above results confirmed the mainly surface chemical structures and Fe coordination composition of MML-CDs, and provided evidence to further investigate its potential effect.

### ROS-scavenging ability of MML-CDs

**Fig. 2:**
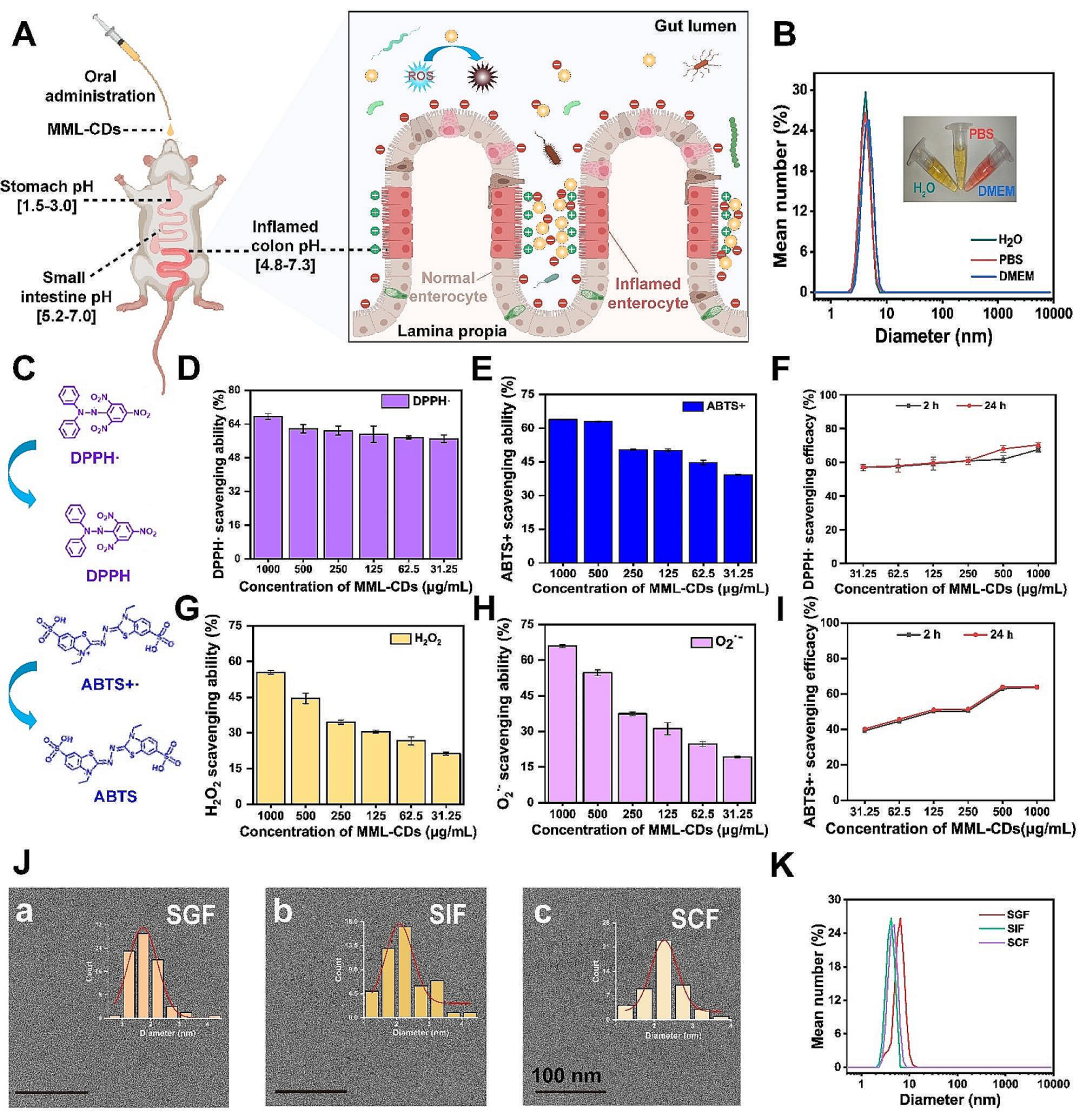
ROS scavenging capacity and GI tract stability of MML-CDs. (**A**) Schematic illustration of oral administration of MML-CDs for treatment of UC. (**B**) DLS assay of MML-CDs in water, PBS and DMEM, respectively. (**C**) Schematic illustration of the ROS scavenging process involving free radicals DPPH· and ABTS+·). (**D**) DPPH· scavenging ability of various concentrations of MML-CDs (*n* = 6). (**E**) ABTS+· scavenging ability of various concentrations of MML-CDs (*n* = 6). (**F**) Time-dependent investigation of DPPH· scavenging ability in the presence of MML-CDs. (**G**) H_2_O_2_ scavenging ability of various concentrations of MML-CDs (*n* = 6). (**H**) O_2_^·−^ scavenging ability of various concentrations of MML-CDs (*n* = 6). (**I**) Time-dependent investigation of ABTS+· scavenging ability in the presence of MML-CDs. (**J**) TEM images of MML-CDs in (**a**) simulated gastric fluid, (**b**) simulated small intestine fluid, and (**c**) simulated colonic fluid, scale bar: 100 nm. (**K**) DLS assay of MML-CDs in different simulated gastrointestinal fluid

The metal coordination of nanoparticles can act as catalytic centers for free radicals and as hydrogen ion donors, thereby further enhancing the scavenging ability of reactive oxygen species (ROS) [41]. The broad-spectrum antioxidant activity of newly developed nanomedicines is one of the key factors in improving ulcerative colitis (UC). Notedly, Fe-doped MML-CDs may provide well catalytic efficiency attributed to the internal electronic environment with more active sites and novel functions. To detect the free radical-scavenging ability, MML-CDs were explored using DPPH· and ABTS+· assays (Fig. 2C). As depicted in Fig. 2D, with increasing concentrations of MML-CDs, the free radical scavenging ability also increased, with approximately 67% of DPPH· being scavenged at a concentration of 1000 µg/mL of MML-CDs. In the presence of MML-CDs at varying concentrations, the characteristic absorption peak of DPPH· radicals at 517 nm gradually decreased after a 2-hour incubation period, and this trend was observed up to 24 h (Fig. S7A and Fig. 2F). Similarly, the ABTS+· scavenging ability of MML-CDs reached up to 64% at 1000 µg/mL, and the UV absorbance of MML-CDs at 730 nm showed a significant decrease (Fig. S7B and Fig. 2I). Additionally, MML-CDs also exhibited well scavenging ability to H_2_O_2_ and O_2_^·−^(Fig. 2G-H). The aforementioned results demonstrate that MML-CDs possess robust antioxidant activities.

To assess the cellular antioxidant capacity of MML-CDs, an H_2_O_2_-induced oxidative damage model was established using Caco-2 cells (Fig. S8A). Cell viability, as measured by the CCK-8 assay, showed no significant cytotoxicity after treatment with various concentrations of MML-CDs for 12 h (Fig. S8B). Conversely, the addition of H_2_O_2_ did not impact cell survival in the presence of MML-CDs (Fig. S8C). During oxidative stress, malondialdehyde (MDA) and ROS levels typically rise in the cellular environment, while antioxidant enzymes are suppressed [42]. A significant reduction in MDA levels was observed between the model group and the MML-CDs group (*P* < 0.001) (Fig. S6D), indicating that MML-CDs have MDA inhibitory activity. Compared to the model group, treatment with MML-CDs effectively enhanced the expression of antioxidant-related mRNAs for Nrf2 and HO-1 (Fig. S6E-F), which may be associated with the enzyme-like activity of MML-CDs [11]. These findings support the conclusion that MML-CDs have potent antioxidant capabilities and provide significant protection against oxidative damage in colon cells.

### The stability evaluation of MML-CDs

The inflamed colon has previously been found to exhibit high tissue permeability and an increased presence of positively charged inflammatory proteins, which facilitates the aggregation of negatively charged nanoparticles (<100 nm) within colonic tissue more effectively than small molecules [43]. In this study, the stability of MML-CDs in various solvents (water, PBS, and DMEM) showed that MML-CDs have a narrow and stable peak with no significant charge difference in different solvent dispersion systems (Fig. 2B and Fig. S9), and no significant changes in their polydispersity index (PDI) and hydrodynamic diameter observed over a 6-day period (Fig. S10). This evidence confirms the high stability of MML-CDs in different solutions.

Subsequently, to investigate the oral potential of MML-CDs, we designed a simulated gastrointestinal environment to evaluate their stability (Fig. 2A). After incubation in simulated gastric fluid (SGF), simulated intestinal fluid (SIF), and simulated colonic fluid (SCF), the appearance of MML-CDs (<10 nm), as assessed by TEM, showed no significant change after a 6-hour mixing period (Fig. 2J and Fig. S11). Meanwhile, based on dynamic light scattering (DLS) assays, MML-CDs in different simulated fluids displayed similar hydrodynamic diameters (Fig. 2K) and Tyndall effects (Fig. S12), suggesting that MML-CDs have good resistance to digestive enzymes and acidic fluids. Moreover, no obvious difference in PDI or ζ-potential measurements of MML-CDs were observed in simulated gastrointestinal fluids (Fig. S13). The complex gastrointestinal environment typically impacts antioxidant activities due to its extreme pH values. However, the antioxidants of MML-CDs still retained over 60% of their DPPH· and ABTS+· scavenging abilities in various simulated fluids, indicating that the antioxidants of MML-CDs can function efficiently in the gastrointestinal environment (Fig. S14). Furthermore, the ζ-potentials of MML-CDs showed no significant differences in negative charge (-3.69, -2.84, and − 2.76 mV) even in SCF within 20 days (Fig. S15), suggesting that MML-CDs could be beneficial for passive targeting to sites of inflammation. Collectively, the results indicated that ultrasmall MML-CDs have excellent oral potential in the treatment of UC due to their high stability, stable negative charge, and antioxidant capacity.

### Biocompatibility of MML-CDs

**Fig. 3:**
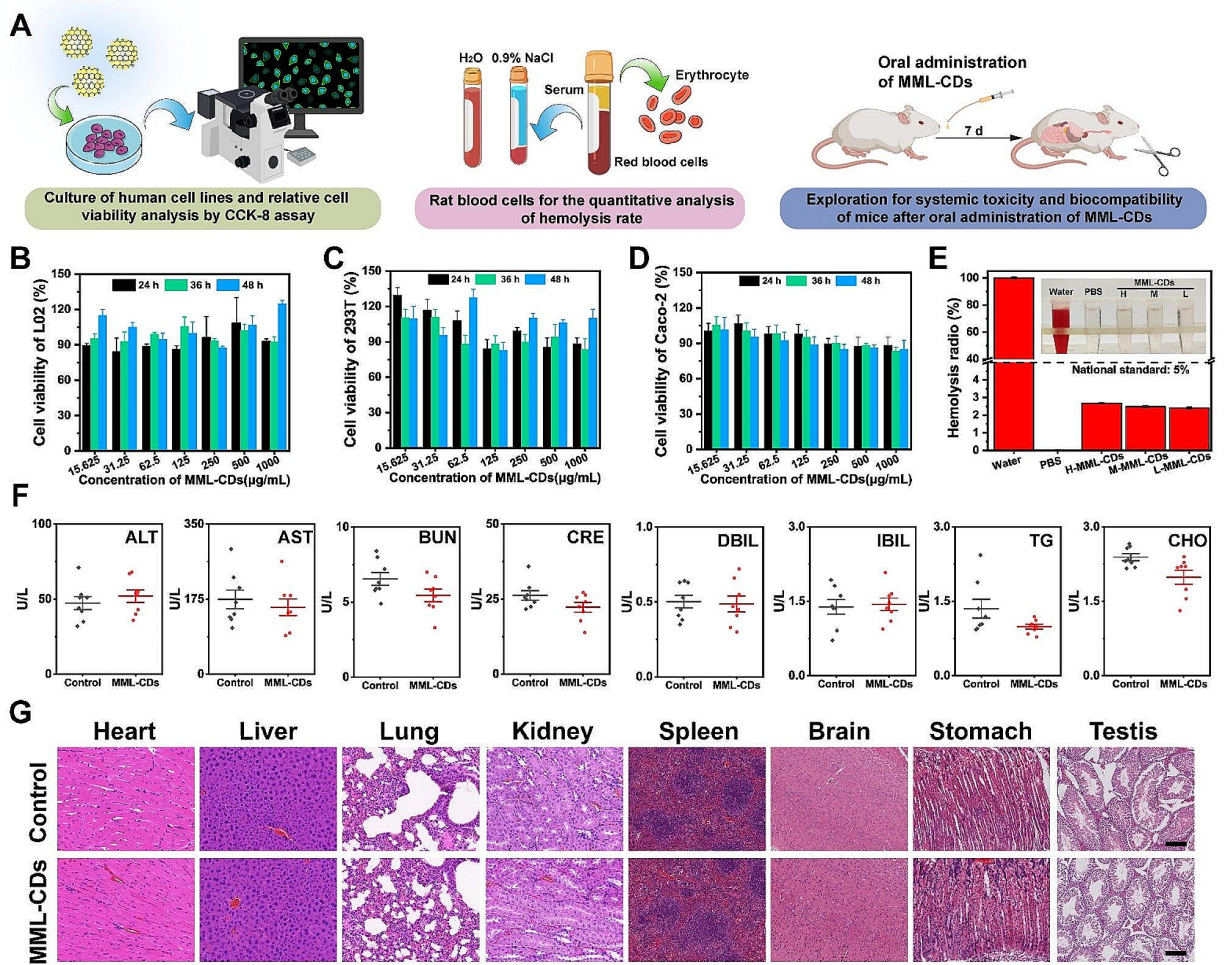
Biocompatibility and biotoxicity analysis of MML-CDs. (**A**) Schematic illustration of the biocompatibility exploration protocols of MML-CDs. (**B**-**D**) Cell viability of MML-CDs on L02, 293T, and Caco-2 cells, respectively. (*n* = 6). (**E**) Hemolysis analysis of MML-CDs (*n* = 6). (**F**) Blood biochemical index (*n* = 6). (**G**) Major organs pathology based on H&E staining after oral administration of MML-CDs, scale bar: 100 μm. Data are presented as mean ± SD and analyzed with two tailed student T test. (^***^P < 0.001, versus control group)

Prior to administering of MML-CDs for the treatment of UC, their overall biocompatibility was assessed through in vitro and in vivo studies. The experimental design details were provided in in Fig. 3A. Furthermore, the cytotoxicity of MML-CDs on L02, 293T, and Caco-2 cells was assessed using the CCK-8 assay, as shown in Fig. 3B-D. All concentration of MML-CDs demonstrated acceptable cytotoxicity over 24, 36, and 48 h. Cellular apoptosis or necrosis typically leads to the fragmentation of the cell membrane, which in turn results in the release of various intracellular biomolecules, such as lactate dehydrogenase (LDH), into the culture medium [44]. This indicated that even the highest concentration of MML-CDs tested (1000 µg/mL) caused no more than 2% LDH release into the medium, suggesting minimal impact on cell membrane integrity (Fig. S16). Moreover, hemolysis assay was conducted to evaluate potential hemolytic effects. MML-CDs at a concentration of 500 mg/mL demonstrated a hemolysis rate below the national standard of 5% (Fig. 5E), indicating high biocompatibility with blood cells [45].

The in vivo toxicity assessment of MML-CDs involved administering healthy BALB/c mice with either PBS or a 500 mg/kg dose of MML-CDs. After a 7-day administration period, comparative analysis revealed no significant differences in body weight or survival rate between the control and MML-CDs-treated groups (Fig. S17). Additionally, blood biochemical parameters of the MML-CDs-administered mice mirrored those of the control group (Fig. 3F). These parameters included alanine aminotransferase (ALT), aspartate transaminase (AST), blood urea nitrogen (BUN), creatinine (CRE), direct bilirubin (DBIL), indirect bilirubin (IBIL), triglycerides (TG), and cholesterol (CHO). Most notably, histological examination of major organs (heart, liver, lung, kidney, spleen, brain, stomach, and teste) from both groups, as shown in Fig. 3G, showed no apparent abnormalities or inflammatory changes. Collectively, these observations pointed towards the high biocompatibility and negligible systemic toxicity of MML-CDs.

### Alleviation of DSS-induced ulcerative colitis by MML-CDs

**Fig. 4:**
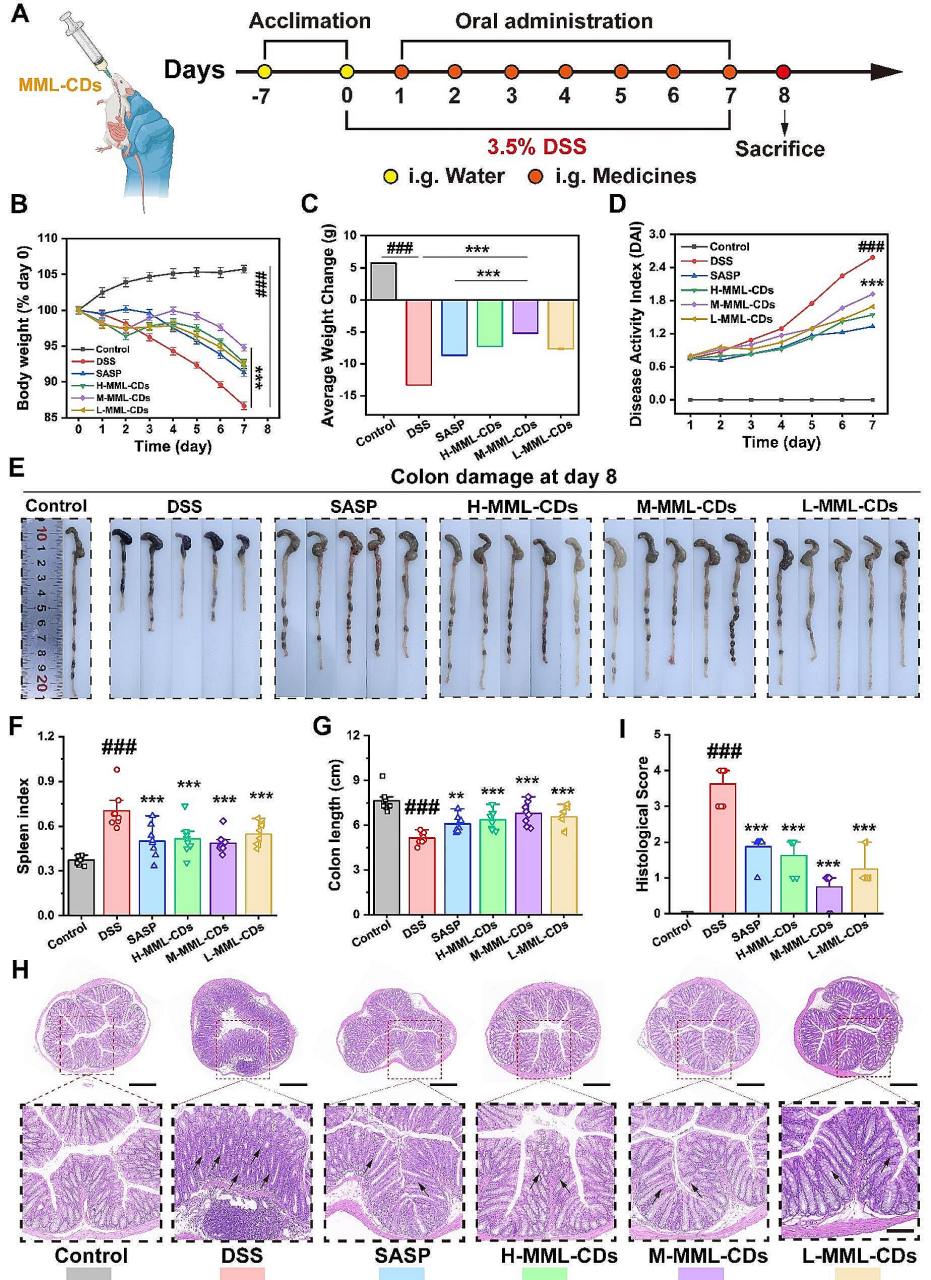
Alleviation of DSS-induced ulcerative colitis by MML-CDs. (**A**) Schematic illustration of the modeling and administration methods. (**B**) Body weight change of different administration groups (*n* = 8). (**C**) Average body weight change of different administration groups (*n* = 8). (**D**) Disease activity index in different administration groups (*n* = 8). (**E**) Images of colons in different administration groups. (**F**) Spleen index of different administration groups (*n* = 8). (**G**) Colon length of different administration groups (*n* = 5). (**H**) Representative images of H&E-stained histological sections of colon slides, 40× magnification is presented on the top (scale bar: 1 cm) and 400× magnification is shown at the bottom (scale bar: 100 μm). **I**) Histological score of different administration groups (*n* = 8). Data are presented as mean ± SD and analyzed with one-way ANOVA followed with Tukey post hoc test, (^###^*P* < 0.001, versus control group; ^*^P < 0.05, ^**^P < 0.01, ^***^P < 0.001 versus DSS model group)

DSS-induced ulcerative colitis in mice is one of the most widely used models due to its high similarity in symptoms and histopathological features to those observed in humans [46, 47]. Our study successfully modeled ulcerative colitis (UC) in mice by administering 3.5% DSS orally for 7 days, consistent with prior reports (Fig. 4A) [48]. According to the biodistribution of DSS, oral gavage was administered at similar times each day, followed by the onset of hematochezia and diarrhea at the indicated time points. Changes in body weight play a critical role in the UC model. As shown in Fig. 4B, all DSS-treated groups showed significant differences compared to the control groups (*P* < 0.001), particularly in the DSS group, which confirmed the successful establishment of the UC mouse model. The weight changes in all MML-CDs dosage groups tended to be similar to those in the sulfasalazine (SASP) treatment group, and all showed improvement compared to the DSS group. By averaging the weight change as depicted in Fig. 4C, the middle dose of MML-CDs was observed to provide better alleviation than the clinical drug SASP (*P* < 0.001). The disease activity index (DAI) scores the severity and development of UC symptoms, including hematochezia, diarrhea, and weight change. The results revealed that DAI measurements were relieved to a certain extent in both MML-CDs and SASP groups (Fig. 4D), while MML-CDs treatment significantly suppressed the diarrhea and hematochezia indices (Fig. S18). Additionally, UC mice treated with MML-CDs showed a reduction in bleeding and diarrhea (Fig. 4E) and improved colon integrity (Fig. 4G), whereas the SASP treatment group exhibited a lesser alleviation effect (*P* < 0.01) compared to the MML-CDs treatment groups (*P* < 0.001). Furthermore, the spleen index in the DSS group was significantly increased, and MML-CDs treatment could alleviate this increase in the DSS treatment group (Fig. 4F).

To further confirm the protective effect of MML-CDs treatment, hematoxylin and eosin (H&E) staining of the colon was performed. Pathological injury was indicated by star marks and scored. As shown in Fig. 4H, DSS treatment induced severe destruction of the villi structure and intestinal crypt necrosis in colon tissue, accompanied by infiltration of neutrophils and other inflammatory cells. In contrast, the results from the drug treatment groups showed that colon structure and inflammatory infiltration were significantly relieved to a remarkable extent in the middle dose of MML-CDs groups, with partial relief observed in the SASP group and the MML-CDs groups with low and high doses (Fig. 4I). Although the exact pathogenesis of ulcerative colitis remains unclear, immune and inflammatory responses within the disrupted colonic barrier structure are undoubtedly involved [43]. The oral administration of different doses of MML-CDs can alleviate these pathological conditions to varying degrees, thereby mitigating the progression of colitis.

### Hemostatic properties of MML-CDs

**Fig. 5:**
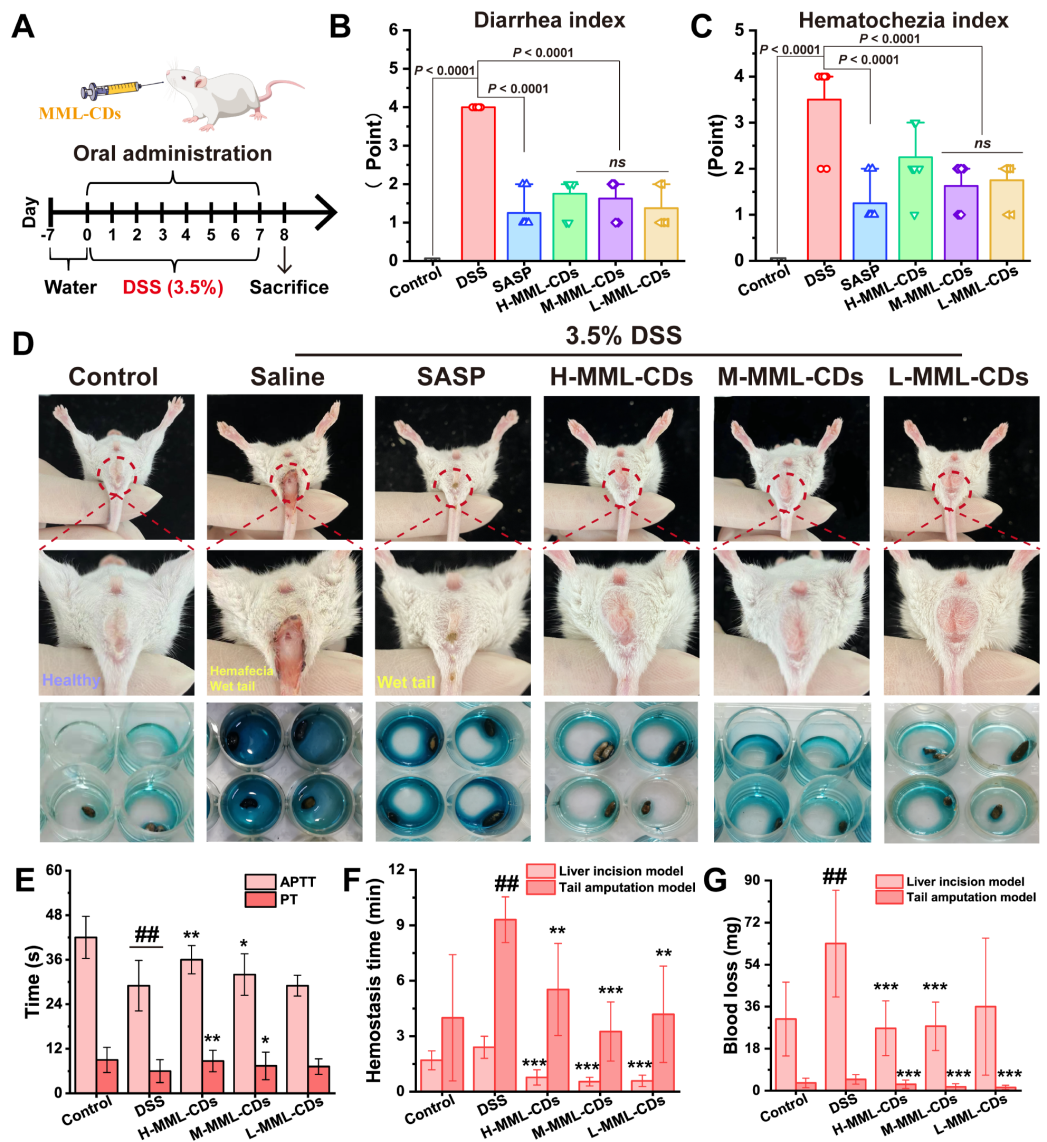
Hemostatic properties of MML-CDs to alleviate UC. (**A**) Schematic illustration of the modeling and administration methods. (**B**) Diarrhea index and (**C**) hematochezia index on day 7 in various groups (*n* = 8). (**D**) Representative photographs of the wet tail and hematochezia on day 7 in various groups. (**E**) Systemic bleeding risk evaluation via measurement of activated partial thromboplastin time (APTT) and prothrombin time (PT). (**F**) Hemostasis time and (**G**) blood loss of liver incision and tail amputation models after administrating of MML-CDs. (*n* = 8) Data are presented as mean ± SD and analyzed with one-way ANOVA followed with Tukey post hoc test. (^###^*P* < 0.001, versus control group; ^*^P < 0.05, ^**^P < 0.01, ^***^P < 0.001 versus DSS model group)

In the progression of ulcerative colitis, symptoms such as intestinal bleeding and diarrhea can exacerbate inflammation and facilitate harmful bacterial infections leading in high treatment costs and prolonged treatment period [30]. The administration of MML-CDs demonstrated significant ability to facilitate coagulation compared to the negative control group, suggesting that MML-CDs may indeed promote blood clotting (Fig. S19A). To further evaluate this ability in vitro, we modeled DSS-induced UC in mice and recorded solutions of diarrhea and hematochezia (Fig. 5A). After treatment with DSS for 7 days, all mice in the ulcerative colitis model developed symptoms, including diarrhea (wet tail) and hematochezia (unclean perianal region and fecal occult blood) (Fig. 5D), primarily due to rapidly proliferating enterocyte death and an acute inflammatory response in the lamina propria caused by a disrupted colon barrier [49]. Additionally, almost all doses of MML-CDs (except the high dose) improved the condition, evidenced by a statistically significant decrease in diarrhea and hematochezia indices in UC mice (Fig. 5B-C). Concurrently, improved red blood cell (RBC) measurements also indicated that MML-CDs could alleviate bleeding on the surface of the colon (Fig. S19B). These experiment results indicated that administration of MML-CDs have treatment potential to improve the hemorrhage and diarrhea of UC in mice.

Previous studies have found that DSS treatment may decrease coagulation and increase the risk of systemic bleeding [50]. The classical hepatic hemorrhage and tail amputation models were performed to assay the hemostatic properties of MML-CDs. The data revealed that the DSS-induced model had prolonged hemostasis times and higher blood loss (*P* < 0.01). Conversely, administration of different doses of MML-CDs effectively improved hemostasis levels (Fig. 5F-G). Coagulation indicators were also utilized to assess systemic bleeding risk. Compared to the control group (Fig. 5E), the DSS group exhibited lower activated partial thromboplastin time (APTT) and prothrombin time (PT), a result of the hypercoagulation state associated with inflammation (both *P* < 0.01). Moreover, administration of high and middle doses of MML-CDs resulted in significantly longer APTT and PT compared to the DSS model group (High dose: *P* < 0.01; Middle dose: *P* < 0.05). During these experiments, we found that MML-CDs could serve as an alleviation strategy in hemostasis during the development of colitis. Recent clinical endoscopy has approved the use of inorganic mineral-based hemostatic powders due to their excellent hemostasis ability by blocking active bleeding sites and absorbing blood [51]. Similarly, MML-CDs may promote coagulation due to their compact structure and excellent water solubility, as well as the doping of Fe ions and the negative charge on their surface, which likely facilitates passive binding with inflamed intestinal segments. The above results confirmed that the hemostatic properties of MML-CDs were benefit to inhibit the development of inflamed intestines.

### Anti-inflammatory and anti-oxidation effects of MML-CDs

**Fig. 6:**
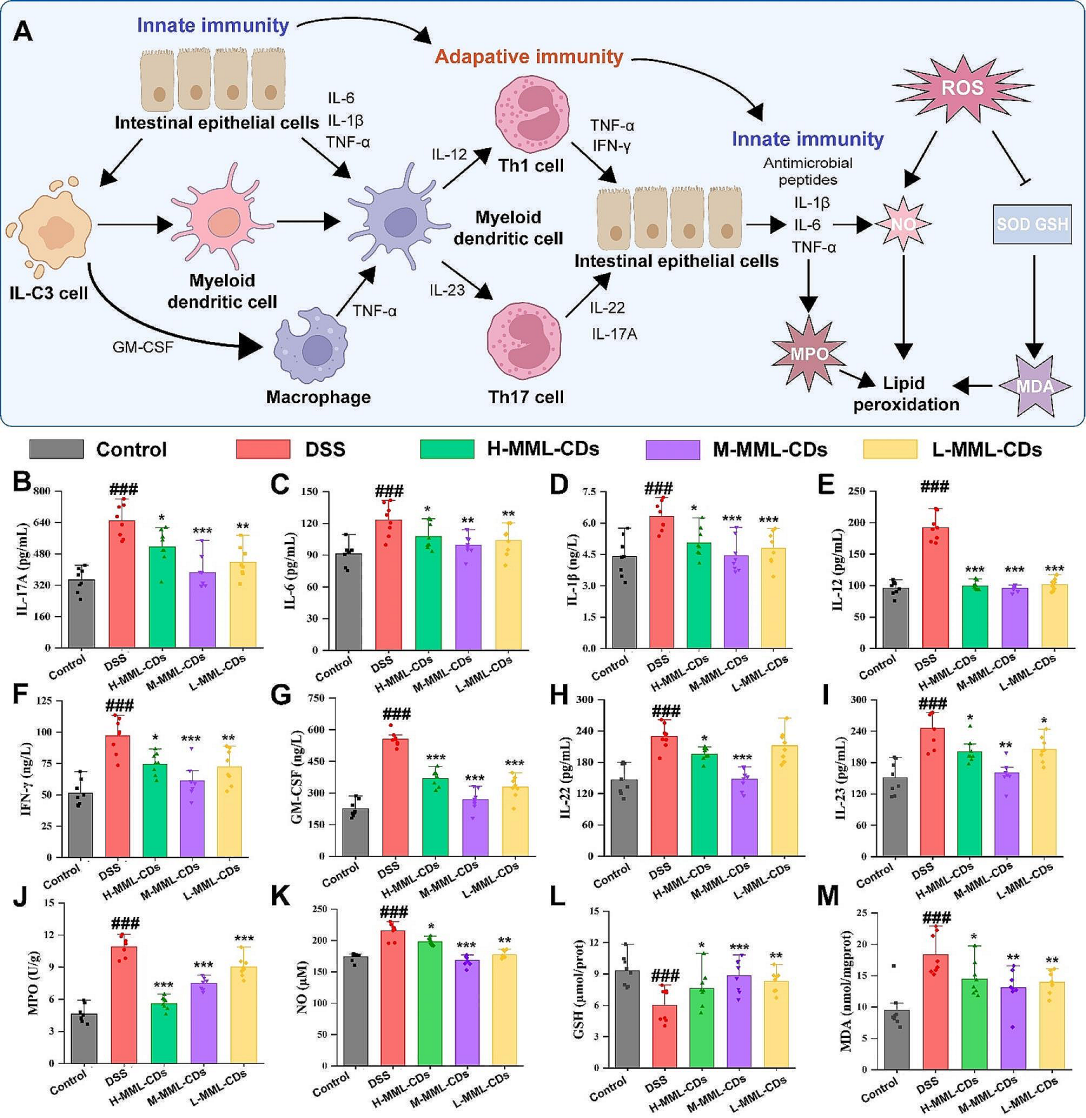
Ulcerative colitis-related proinflammatory cytokine and oxidative stress indicator expression in UC mice after oral administration of MML-CDs. (**A**) Schematic illustration of key cells and mediators in the transition from innate to adaptive immunity in ulcerative colitis. Proinflammatory cytokine levels of (**B**) IL-17 A, (**C**) IL-6, (**D**) IL-1β, (**E**) IL-12, (**F**) IFN-γ, (**G**) GM-CSF, (**H**) IL-22, and (**I**) IL-23 in colon tissues. Oxidative stress levels of (**J**) MPO, (**K**) NO, (**L**) GSH, and (**M**) MDA. (*n* = 8) Data are presented as mean ± SD and analyzed with one-way ANOVA followed with Tukey post hoc test, while the comparison of non-normal distributed data was evaluated by the Kruskal-Wallis H test. (^###^*P* < 0.001, versus control group; ^*^P < 0.05, ^**^P < 0.01, ^***^P < 0.001 versus DSS model group)

To further evaluate whether MML-CDs administration could downregulate the inflammatory response in ulcerative colitis (UC) lesions in vivo, several key UC-related cytokines were assayed. There are several key cells and mediators involved in the development of ulcerative colitis [52]. As depicted in Fig. 6A, myeloid dendritic cells are activated by key cytokines produced by immune cells, and granulocyte-macrophage colony-stimulating factor (GM-CSF) also activates macrophages to release inflammatory cytokines. Activated dendritic cells present antigens and release substances, including interleukin-12 (IL-12) and interleukin-23 (IL-23), which have been confirmed to trigger the formation of Th17 and Th1 helper T cells [34]. Subsequently, these T cells release factors such as interleukin-17A (IL-17A), interferon-γ (IFN-γ), and interleukin-22 (IL-22), that stimulate colonic epithelial cells to produce antimicrobial peptides, chemokines, and proinflammatory cytokines (tumor necrosis factor-α (TNF-α), interleukin-1β (IL-1β), and interleukin-6 (IL-6)). These mediators are related to the proinflammatory disease cycle by shaping the inflammatory infiltrate and promoting granulocyte aggregation [53]. The protein levels of GM-CSF, IL-17 A, IL-1β, IL-6, TNF-α, IFN-γ, IL-23, and IL-12, as determined by ELISA (Fig. 6B-I, and Fig. S20), were significantly downregulated following MML-CDs administration.

Meanwhile, the pathogenesis of ulcerative colitis is also caused by the inactivation of antioxidant enzyme activity and the accumulation of reactive oxygen species (ROS), as an imbalanced ROS homeostasis contributes to increased lipid peroxidation and exacerbated inflammation expression [16]. With the progression of inflammatory reactions in UC, antioxidant enzymes (e.g., glutathione reductase (GSH)) are progressively consumed, and the levels of malondialdehyde (MDA) and nitric oxide (NO) increase. Furthermore, the infiltration of neutrophils also promotes an increased level of myeloperoxidase (MPO). As revealed by the in vitro results on oxidative stress, MML-CDs administration promoted the downregulation of MDA and MPO expression (Fig. 6J-K), as well as increase the expression of GSH (Fig. 6M). These data confirm that MML-CDs ameliorate the inflammatory infiltrate in ulcerative colitis by inhibiting the inflammatory response and reducing oxidative stress.

### Repair of gut barrier function by MML-CDs

Tight junction (TJ) proteins are critical factors for maintaining the integrity of the colonic mucosal barrier function [53]. The development of colitis invariably leads to a decrease in TJs, which compromises the colonic mucosal barrier and renders it susceptible to harmful bacterial infections and recurrent inflammation. Additionally, claudin proteins can reduce the permeability to positively charged cations due to their electrostatic interactions [54]. This property potentially enhances the electrostatic interaction between TJs and negatively charged MML-CDs, thereby facilitating the function of surface-active groups from their precursors at the tight junction location.

**Fig. 7:**
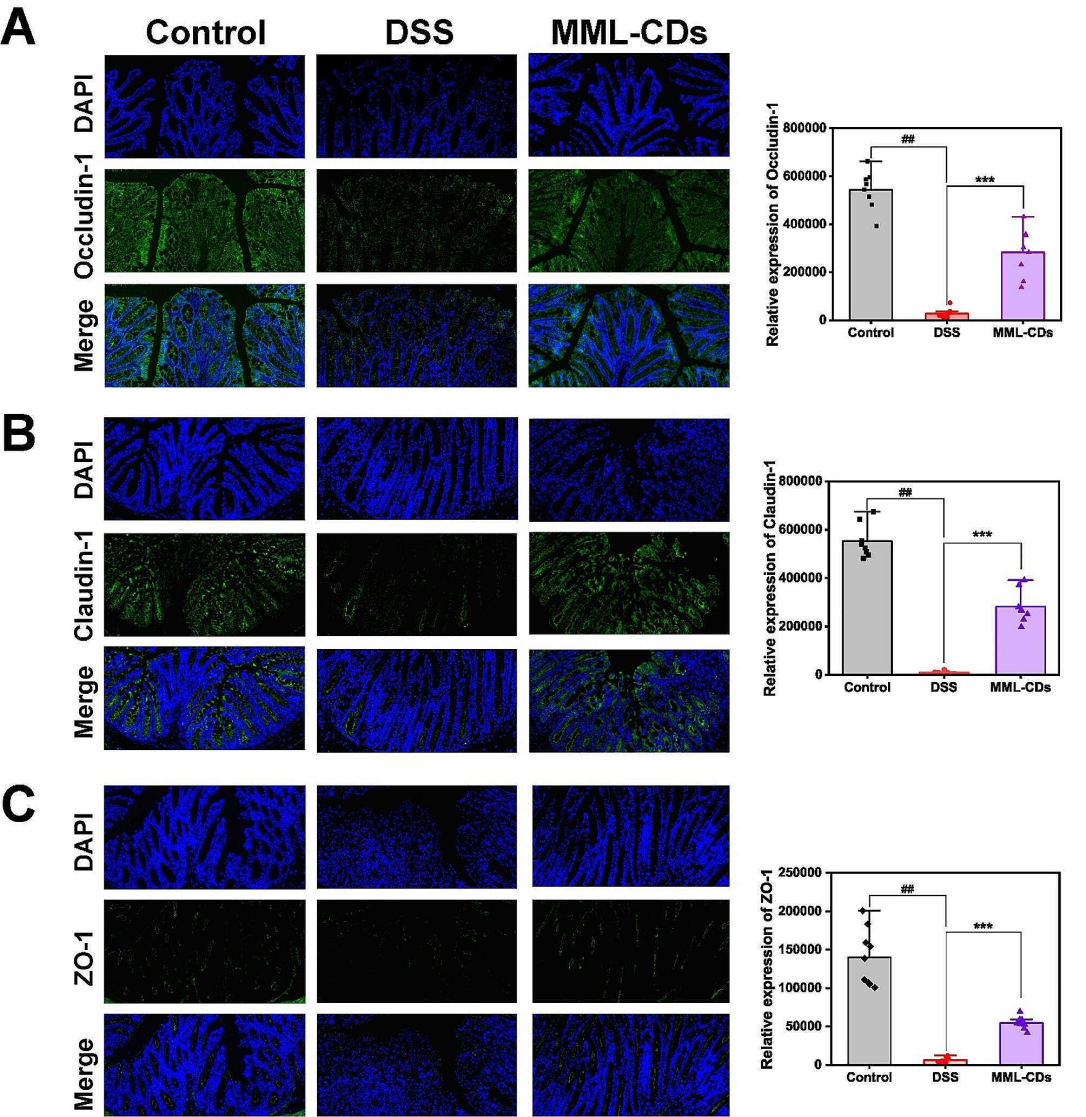
Repair of gut barrier function by MML-CDs. Fluorescence images and relative expression of (**A**) Occludin-1, (**B**) Claudin-1, and (**C**) ZO-1 in the colon tissues, respectively. Data are presented as mean ± SD and analyzed with one-way ANOVA followed with Tukey post hoc test. (^##^*P* < 0.01, versus control group; ^***^P < 0.001 versus DSS model group)

To assess the expression of claudin-1, occludin-1, and ZO-1 in the colonic region, we performed immunofluorescence on sections of colonic tissue. The protein expression levels of claudin-1, occludin-1, and ZO-1 were significantly downregulated in colonic mucosal sections after treatment with DSS for 7 days, suggesting a disruption in the colonic barrier integrity (Fig. 7A-B). In contrast, the results of claudin-1 and occludin-1 staining from the MML-CDs treatment group showed larger mean areas, indicating that MML-CDs enhanced the expression of these TJ proteins compared to the DSS-treated group. Similarly, the expression of the ZO-1 protein was upregulated in the mucosal crypts following MML-CDs administration (Fig. 7C). The carbon dots (CDs) extracted from *Semen pruni persicae* and *Carthamus tinctorius* were used to construct simplified virtual models of CDs, which revealed possible interaction forces between CDs and TJs, such as hydrogen bonding, van der Waals forces, and π–π stacking [31]. This suggests that bonding between CDs and TJs may promote the upregulation of TJ expression. The actual mechanism underlying this interaction will need to be verified by robust research techniques in future studies.

### Regulation of intestinal flora by MML-CDs

**Fig. 8:**
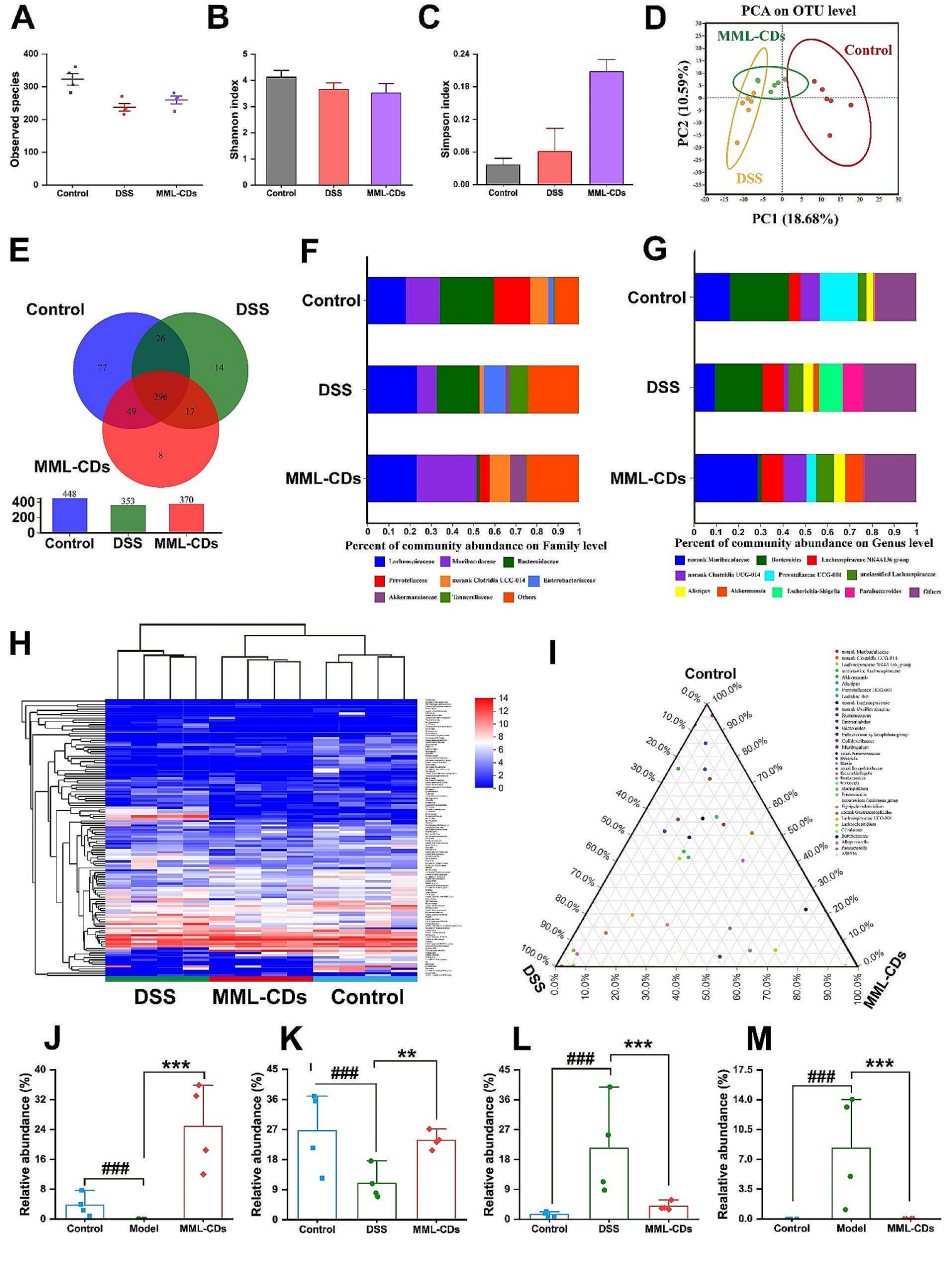
Regulation of intestinal flora by MML-CDs. (**A**-**C**) Estimation of microbial community observed operational taxonomic units (OTU) richness and α-diversity (Shannon and Simpson index). (**D**) PCA plot illustrating the intestinal microbiota β-diversity. (**E**) Venn diagram of shared and unique species on OTU level. (**F**) Relative abundance of top 20 species at Family level. (**G**) Relative abundance of top 20 species at Genus level. (**H**) Heatmap of the relative abundance of genus-level taxonomy for each mouse. (**I**) The distribution proportion of the dominant species in the intestinal flora of mice in the control, DSS and MML-CDs group based on Species taxonomic level. Relative abundance of MML-CDs on (**J**) *Prevotellaceae UCG 001*, (**K**) *norank munibaculaceae*, (**L**) *Bacteroides* and (**M**) *Escherichia shigella* (*n* = 4). Data are presented as mean ± SD and analyzed with one-way ANOVA followed with Tukey post hoc test. (^###^*P* < 0.001, versus control group; ^**^P < 0.01, ^***^P < 0.001 versus DSS model group)

Recent studies have provided growing evidence that the intestinal microbiota has a direct impact on the pathogenesis of ulcerative colitis [55]. Alpha diversity analysis at the operational taxonomic unit (OTU) taxonomic level was performed by measuring observed species (Fig. 8A and Fig. S21), Shannon (Fig. 8B), Simpson (Fig. 8C), and Chao indexes (Fig. S22). These results determined that MML-CDs could enhance bacterial richness and diversity. Principal component analysis (PCA) revealed that MML-CDs were closer to the control group than to the model group, indicating that MML-CDs have a different community composition from the model group (Fig. 8D). A Venn diagram displayed the different species numbers (OTUs) between the control, DSS, and MML-CDs groups (448, 353, and 370, respectively), suggesting that MML-CDs administration may promote an increase in species numbers (Fig. 8E).

To evaluate changes in the intestinal microbiota, the community composition at the Phylum, Family, and Genus levels was analyzed (Fig. S23). Among the top 20 species at the Family level (Fig. 8F), compared to the control group, the proportion of community abundance for beneficial bacteria (such as *Lachnospiraceae*, *Bacteroidaceae*, *Enterobacteriaceae*, and *Tannerellaceae*) significantly increased, while the proportion for harmful bacteria (e.g., *Muribaculaceae*, *Prevotellaceae*, unclassified *Clostridiales UCG-014*, and *Akkermansiaceae*) also decreased. The community composition at the genus level showed similar results (Fig. 8G). Subsequently, the heatmap results indicated that the community composition of MML-CDs was similar to that of the control group, suggesting that MML-CDs can improve the community composition of the intestinal microbiota (Fig. 8H). Additionally, the distribution proportion at the genus taxonomic level was depicted using a circular diagram (Fig. S24). Furthermore, a ternary phase diagram of species composition and distribution showed a greater presence of beneficial bacterial groups between the DSS group and the MML-CDs group (Fig. 8I).

Linear discriminant analysis (LDA) effect size (LEfSe) is an analytical tool that used for identifying and interpreting microbial biomarkers. It can be employed to assess species with statistical differences among various groups, and 33 main biomarkers were identified with high LDA scores (scores > 3) (Fig. S25). Compared to the control group, the families unclassified *Muribaculaceae*, *Peptococcus*, and *Anaerofustis* were relatively more abundant. However, *Bacteroides* and *Escherichia-Shigella* were relatively more abundant at the genus level in the DSS group than in other groups, confirming their role as the main pathogens causing an imbalance in the intestinal microbiota [56]. Meanwhile, MML-CDs demonstrated effective regulation of the intestinal microbiota (Fig. S26). Indeed, MML-CDs could significantly suppressed the main pathogenic bacteria and upregulate the abundance of beneficial bacterial genera (such as unclassified *Muribaculaceae* and *Prevotellaceae UCG-001*) (Fig. 8J-M and Fig. S27). In addition, RDA analysis, Spearman analysis, and 16 S rRNA gene amplicon sequencing analysis were performed to explore the interaction between MML-CDs and intestinal flora (Fig. S28). Prior studies have confirmed that metal-doped carbon dots (including Fe-doped carbon dots) often exhibit potent antibacterial activity in vitro [57, 58], which could explain the non-significant increase in observed species with MML-CDs. Thus, MML-CDs demonstrated well regulation to intestinal flora of UC mice, which may be the potential mechanism to alleviate UC by MML-CDs.

## Conclusion

Overall, MML-CDs exhibited ultrasmall nano-size and a polyphenolic structure doped with Fe ions, possessing remarkable ROS scavenging ability, excellent gastrointestinal stability and highly biocompatibility. Our study found that the therapeutic effects of MML-CDs were confirmed to be superior to first-line clinical drug SASP. MML-CDs efficiently penetrated colonic barrier and maintained ROS scavenging capability in colon environment, which enhanced hemostasis ability, alleviated inflammation reactions and oxidative stress, and repaired colonic barrier damage. The administration of MML-CDs could modulate the diversity and composition of gut microbiota to improve intestinal microenvironment. Our work provided a valuable healthcare potential using MML-CDs treatment for further treatment strategy for ulcerative colitis.

## Methods

### Materials

Magnetite (MA, Ci-shi, Batch No.:190511003) and Medicated Leaven (ML, Shen-qu, Batch No.:210718008) were purchased from Beijing Qiancao Traditional Chinese Medicine Co. Ltd. (Beijing, China). Dextran Sulfate Sodium Salt (DSS, Mw: 36–50 kDa) was procured from MP Biomedicals, Inc. (Irvine, CA, USA). Dialysis membranes (MWCO:1 kDa) were obtained from Beijing Ruida Henghui Technology Development Co., Ltd. (Beijing, China). Haemocoagulase (HC) for injection was purchased from Jinzhou Ahon Pharmaceutical Co., Ltd. (Liaoning, China). Proteinase and phosphatase inhibitors and the BCA assay kit were purchased from Shanghai Beyotime Biotechnology Co., Ltd. (Shanghai, China). Enzyme-linked immunosorbent assay (ELISA) kits were purchased from Jiangsu Kete Biotechnology Co., Ltd. (Jiangsu, China). Other experiment materials were obtained from Sinopharm Chemical Reagents Beijing (Beijing, China). All the experiments were performed utilizing deionized water (DW).

### Preparation of MML-CDs

MML-CDs were synthesized by one-step hydrothermal method. The fine powders of MA (25 g) and ML (50 g) were mixed and dispersed in 1000 mL DW, following with boiling at 100℃ three times for 1 h in thermostatic water bath. The coarse solution was firstly filtered by Büchner funnel, and the obtained filtrate was further filtered through 0.22 μm cellulose acetate membrane. The resulting solution was purified by dialyzing with 1000 Da dialysis membrane for 72 h until the color of solution no longer changes. The MML-CDs solution was obtained after several times of centrifugation to remove any unreacted residue, as well as the filtered solution was freeze-dried and stored in a refrigerator at 4 °C.

### Characterization of MML-CDs

The morphology and size distribution of MML-CDs was observed by transmission electron microscope (TEM, 100 kV, Talos F200X-G2, USA) and high-resolution transmission electron microscope (HRTEM, 200 kV, JEOL, Tokyo, Japan). The hydrodynamic diameter distribution, polymer dispersity index (PDI) and ζ-potential of the as-prepared samples were measured by Malvern particle size analyzer (Malvern Zetasizer Nano ZS 90, UK). X-ray diffractometer (XRD, D8-Advanced X-ray diffractometer, Bruker AXS, Karlsruhe, Germany) was utilized to characterize the crystalline structures of MML-CDs. Ultraviolet-visible (UV-vis) absorption spectra were obtained using a UV-vis spectrophotometer (CECIL, Cambridge, UK). The fluorescence (FL) spectra and fluorescence dependence were carried out by a fluorescence spectrophotometer (F-4500, Tokyo, Japan). Fourier transform infrared (FT-IR) spectrometer was applied to ensure the surface groups of MML-CDs with ranging 400 to 4000 cm^− 1^ (Thermo Fisher, Fremont, CA, USA). The element composition and elemental analysis of MML-CDs utilized X-ray photoelectron spectroscopy (XPS, ESCALAB 250Xi, Thermo Fisher Scientific, Fremont, CA, USA) with a mono Al Kalpha 150 W X-ray source (200 eV for the survey; 30 eV for high resolution scans). Thermogravimetric analysis (TGA, SDT-Q600 thermal analyzer) was used to assess the mass percentage of iron and associated organic components. BET surface area analysis (Quantachrome Autosorb IQ, USA) was performed to evaluate the specific surface area of MML-CDs.

### Free radical scavenging tests

The free radical scavenging capacity of MML-CDs was performed for DPPH· and ABTS+·. In detail, DPPH· (50 µM), as a stable nitrogen center free radical, was added into different amounts of MML-CDs solution (1000, 500, 250, 125, 62.5 and 31.25 µg/mL). With the removal of free radicals, there was an absorption peak change at 517 nm. Similarly, ABTS+· was obtained through mixing 0.8 mL ABTS (7 mM) and 1 mL potassium persulfate (K_2_S_2_O_8_, 2.45 mM) overnight in the dark, and incubated with MML-CDs as the same concentrations of MML-CDs in DPPH· scavenging experiment. The absorbance values of ABTS+· at 738 nm were recorded to quantify the free radical scavenging ability of MML-CDs. The absorbance of DPPH· and ABTS·+ with no MML-CDs were separately served as positive control. Moreover, UV-vis absorption spectra of above solutions of DPPH· and ABTS+· were monitored to exhibit the free radical scavenging abilities. Radical scavenging capacity (%) was calculated as Eq. (1):

1 $$\eqalign{Radical\,scavenging\,capacity\,\left(\%\right)= {{{\rm{O}}{{\rm{D}}_{{\rm{Positive}}\,{\rm{control}}}}{\rm{ }} - {\rm{ O}}{{\rm{D}}_{{\rm{samples}}}}} \over {{\rm{O}}{{\rm{D}}_{{\rm{Positive}}\,{\rm{control}}}}}} \times 100 \cr}$$

### Cell culture

Cell lines and cell culture: Human liver cells (L02), human renal epithelial cells (293T) and human colorectal adenocarcinoma cells (Caco-2) were provided by National Experimental Cell Resource Sharing Platform (Beijing, China) and cultured in DMEM (Gibco, USA) containing 20% fetal bovine serum (FBS, Corning, USA) and 1% penicillin-streptomycin (Gibco, USA) under the condition of a humidified atmosphere (5% CO_2_) at the temperature of 37 ℃.

### ROS scavenging activity evaluation of MML-CDs

Caco-2 cells were chosen to model H_2_O_2_-induced oxidative damage. At first, after treated with DMEM or MML-CDs (31.25 µg/mL) for 24 h, different concentrations of H_2_O_2_ (0-200 µM) were incubated with Caco-2 cells at a density of 1 × 10^5^ cells/well in 96-well plates for 8 h, and the CCK-8 assay was used to detected the cytotoxicity of MML-CDs in the presence of various concentrations of H_2_O_2_. Secondly, Caco-2 cells were seeded into 12-well plate for 24 h and incubated with MML-CDs for 24 h, then were incubated with 200 µmol/L H_2_O_2_. Furthermore, the cells were collected, detected MDA levels and mRNA expression of Nrf2 and HO-1 (Primers details in Table S1). The cell viability (%) was calculated in the following Eq. (2):

2 $$\:Cell\:viability\:\left(\%\right)=\frac{{\text{O}\text{D}}_{\text{S}\text{a}\text{m}\text{p}\text{l}\text{e}}\:-\:{\text{O}\text{D}}_{\text{b}\text{l}\text{a}\text{n}\text{k}}}{{\text{O}\text{D}}_{\text{C}\text{o}\text{n}\text{t}\text{r}\text{o}\text{l}}-\:{\text{O}\text{D}}_{\text{b}\text{l}\text{a}\text{n}\text{k}}}\times\:100$$

### Simulated gastrointestinal stability of MML-CDs

Firstly, the simulated gastric fluid, small intestine fluid and intestinal fluid was obtained as the previous papers reported [59]. Then, MML-CDs with an equivalent concentration of 1 mg/mL were co-incubated in the simulated gastric fluid, simulated intestinal fluid and simulated colonic fluid, respectively. Meanwhile, TEM and DLS assay were separately performed to observe the changes of morphology, hydrodynamic diameter distribution and ζ-potential in simulated gastrointestinal fluids. Meanwhile, the size distribution and ζ-potential of MML-CDs were measured in water, PBS (pH 7.4), and DMEM by DLS profiles to test its stability in different dispersing medium.

### Biocompatibility of MML-CDs

The cytotoxicity assays of MML-CDs on L02, 293T, and Caco-2 cells were investigated by CCK-8 assay. Meanwhile, the hemolysis assay of MML-CDs was measured with fresh rat red blood cells. Furthermore, the biocompatibility in vivo was assessed by serum biochemical experiment and Hematoxylin-Eosin Staining (H&E). All experiment details were recorded in the Supporting Information.

### Animals

Male BALB/c mice (7-week-old) were purchased from SiPeiFu Biotechnology Co., Ltd (Beijing, China). Mice were acclimatized in a clean-grade animal room under 12 h/ 12 h light and dark cycle at a constant temperature (23 ± 2 ℃) and humidity of 50 ± 10% and were provided with standard chow and water ad libitum. All experimental procedures and animal care were approved by the National Laboratory Animal Welfare Guidelines approved by Beijing University of Chinese Medicine (BUCM-4-2021-102502-4021).

### DSS-induced ulcerative colitis induction and treatment

All BALB/c mice were randomly divided into six groups (*n* = 6) after acclimating for 7 days: Control group (DW), DSS group (DW), SASP group (SASP, 500 mg/kg), High dose of MML-CDs (H-MML-CDs, 250 mg/kg) group, Meddle dose of MML-CDs (M-MML-CDs, 125 mg/kg) and Low dose of MML-CDs (L-MML-CDs, 62.5 mg/kg). The control group was given normal DW while the rest were given 3.5% DSS (w/v) to induce colitis, as well as fresh DSS drinking water was prepared and replaced the old DSS drinking water every 2 days. During modeling for 7 days, body weight change, stool consistency, and the occurrence of diarrhea were recorded, and the stool was collected. Briefly, the clinical disease activity index (DAI) was scored to compare UC severity of treatment, which are based on human UC characteristic symptoms normally. The DAI score was taken as the sum of the individual scores for each feature, graded on a scale ranging 0 to 4 as follows: 0 (no symptoms), 1 (mild), 2 (moderate), 3 (severe) and 4 (very severe), for each of the following three symptoms: (1) Weight loss, (2) Stool condition and (3) Occult or gross bleeding. The parameters, grades and scores of DAI were detailed in Table S2. At the end of the experiment in 8 d, all animals were sacrificed by taking blood from the eyeball. The blood, colon, spleen and stool were collected to perform in next experiments.

### Hemostatic properties tests

The activated partial thromboplastin time (APTT) and Prothrombin time (PT) assay are important monitors of the intrinsic coagulation system, while a significant increase in platelets also marks an increase in clotting ability [60]. Clinical APTT, PT and blood platelet (BP) tests were conducted using an automatic coagulation analyzer (RAC-030, Rayto, China). Meanwhile, after modeling and treatment for 7 days, the hemostatic effects in DSS-induced mice were evaluated by tail amputation and liver incision assay, with process details referring to previous research [61].

### H&E and immunofluorescence staining

Tissue samples were harvested and fixed in 4% paraformaldehyde overnight, and next dehydrated and embedded in paraffin blocks. Then, 5 μm histological sections were cut and stained with H&E trichrome. For immunofluorescence staining, the paraffin slices were firstly deparaffinized by 3% hydrogen peroxide in 10 min, then blocked by blocking buffer for an hour, and next incubated in primary antibody for 12 h at 4℃, including ZO-1 at 1:1000, Claudin-1 at 1:500 and Occludin-1 at 1:1000. The slices were immersed in the dilution buffer with goat anti-rabbit secondary antibody for 50 min at 20℃ and next rinsed with PBS 3 times, counterstaining with DAPI for 10 min and mounting. The expression of TJs was observed by fluorescent microscope.

### In vivo anti-inflammation property

After homogenizing by pre-cooled 0.9% normal saline, the suspensions of the colon samples were centrifuged at 3000 rpm for 10 min at 4℃ to sperate supernatant and was performed in the next assay. The activity of myeloperoxidase (MPO) was evaluated by colonic MPO assay kit. Moreover, the concentration of TNF-α, IL-12, IL-22, IL-23, IL-6, IL-1β, IL-17 A, GM-CSF and IFN-γ in colon tissue were assayed using commercial ELISA kits.

### In vivo antioxidant property

The colon tissues preserved in the − 80 ℃ were homogenized with PBS on ice and then centrifuged at 10,000 rpm for 15 min. The supernatants were collected to calculate the content levels of MPO, NO, MDA and GSH using the respective kits in accordance with the manufacture’s instruction.

### Gut microbial analysis

The Microbial DNA in each treatment group was extracted from mice fecal samples using the E.Z.N.A.^®^ Soil DNA Kit (Omega Bio-tek, Norcross, GA, USA) according to the manufacture’s instruction, and next steps of the experiment were supported in the Supporting Information.

### Statistical analysis

The SPSS 13.0 (IBM, USA) software was used for all statistical analysis, and all results were presented as means ± SD (standard deviations). Data between two groups were assessed using independent Student’s t test (two-tailed). Data in multiple groups were calculated by one-way analysis of variance (ANOVA) followed by Tukey post hoc test. Additionally, the comparison of non-normal distributed data was evaluated by the Kruskal-Wallis H test. In this study, ^*^P < 0.05, ^**^P < 0.01 and ^***^P < 0.001 were considered statistically significant.

### Electronic supplementary material

Below is the link to the electronic supplementary material.

Supplementary Material 1

## Data Availability

No datasets were generated or analysed during the current study.
