# Supplementary material for: Natural medicines-derived carbon dots as novel oral antioxidant administration strategy for ulcerative colitis therapy
Source: J Nanobiotechnology. 2024 Aug 27;22:511. doi: 10.1186/s12951-024-02702-2 (PMC11348712; doi:10.1186/s12951-024-02702-2)
Supplement: Supplementary file 1 — Supplementary Material 1 [file 12951_2024_2702_MOESM1_ESM.docx]

**Supplementary Information**

**Natural medicines-derived carbon dots as novel oral antioxidant administration strategy for ulcerative colitis therapy**

Tong Wu^1#^, Xue Bai^2#^, Yue Zhang^3#^, Er-tong Dai^4^, Jin-yu Ma^2^, Cai Yu^5^, Chen-xin He^2^, Qian-nan Li^6^, Ying-xin Yang^2^, Hui Kong^2^, Hui-hua Qu^2,7*^, Yan Zhao^2*^

*^1^ School of Chinese Materia Medica, Beijing University of Chinese Medicine, Beijing, 100029, China*

*2 School of Traditional Chinese Medicine, Beijing University of Chinese Medicine, Beijing, 100029, China*

*3* *School of Life Science, Beijing University of Chinese Medicine, Beijing, 100029, China*

*4 Qingdao Eighth People's Hospital, Qingdao, 266100, China*

*5 Department of Endocrine, Beijing Daxing District Hospital of Integrated Chinese and Western Medicine, Beijing, 100163, China*

*6 Department of Traditional Chinese Medicine, Beijing Daxing District Hospital of Integrated Chinese and Western Medicine, Beijing, 100163, China*

*7 Centre of Scientific Experiment, Beijing University of Chinese Medicine, Beijing, 100029, China*

# These authors contributed equally.

***Correspondence:** [quhuihuadr@163.com](mailto:quhuihuadr@163.com) (Huihua, Qu); [zhaoyandr@163.com](mailto:zhaoyandr@163.com) (Yan,Zhao).

1. **Experiment detail**

**Simulated gastrointestinal stability of MML-CDs**

At first, the simulated gastric fluid (SGF) was prepared with 50 mM of NaCl and 3.2 mg·mL^-1^ of pepsin at pH=1.2 ± 0.3. The simulated intestine fluid (SIF) was obtained with 12.5 mg·mL^-1^ of bile salt and 8 mg·mL^-1^ trypsin from porcine pancreas in PBS (10 mM, pH=7.4). Additionally, the simulated colon fluid (SCF) was prepared with 12.5 mg·mL^-1^ of bile salt and 8 mg·mL^-1^ trypsin from porcine pancreas in PBS (10 mM, pH=8.2). Next, MML-CDs (equivalent concentration: 1 mg/mL) were incubated in the SGF, SIF and SCF for 6 h. The mixture was collected and centrifuged to separate supernatant for TEM, DLS assay, and ζ-potential measurement.

**Cell lines, cell culture, and cytotoxicity tests**

Human liver cells (L02), human renal epithelial cells (293T) and human colorectal adenocarcinoma cells (Caco-2) were provided by National Experimental Cell Resource Sharing Platform (Beijing, China) and cultured in DMEM (Gibco, USA) containing 20% fetal bovine serum (FBS, Corning, USA) and 1% penicillin-streptomycin (Gibco, USA) under the condition of a humidified atmosphere (5% CO_2_) at the temperature of 37 ℃. Cells were cultured in 96-well plates with 1×10^5^/mL. After seeded for 24 h, 100 μL medium containing different concentrations of MML-CDs (1000, 500, 250, 125, 62.5, 31.25 and 15.625 μg/mL) were added to wells and co-cultured for 24 h, 36 h and 48 h. Cytotoxic effects of MML-CDs in different cells were examined by CCK-8 assay according to the manufacturer’s instructions, and the OD of respective wells was recorded at 450 nm using a microplate reader (Biotek, USA). The cell viability (%) was calculated in the following Equation (1):

$Cell viability \left( \% \right)=\frac{\mathrm{OD}_{\mathrm{Sample}} - \mathrm{OD}_{\mathrm{blank}}}{\mathrm{OD}_{\mathrm{Control}}- \mathrm{OD}_{\mathrm{blank}}}\times100$ (1)

**Hemolysis Assay**

The hemolysis assay was tested using fresh rat blood cells. Different concentrations of MML-CDs were mixed with red blood cells to a final red blood cell concentration of 4%. The DW was the positive control, and PBS was the negative control. After incubated for 4 h at 37 ℃, the absorption of supernatant was read at 570 nm by microplate reader. The Equation (2) was used to calculate the hemolysis rate:

$Hemolysis rate \left( \% \right)=\frac{\mathrm{OD}_{\mathrm{Sample}} - \mathrm{OD}_{negative control}}{\mathrm{OD}_{Positive control}- \mathrm{OD}_{negative control}}\times100$ (2)

**Biocompatibility assay**

Health BALB/c mice were intravenously administrated with PBS (Control group) and MML-CDs (MML-CDs group, i.g, 300 mg/kg per 24 h) for 7 days. At 8 d, the blood was obtained for blood routine and biochemical analysis. And the major organs were collected for H&E staining.

**Gut microbial analysis**

Microbial DNA in each treatment group was extracted from mice fecal samples using the E.Z.N.A.® Soil DNA Kit (Omega Bio-tek, Norcross, GA, USA) according to the manufacture’s instruction. The final quantity and quality of DNA using Nanodrop and 1.2% agarose gel electrophoresis, respectively, then the amplification of V3-V4 hypervariable regions of the rRNA genes were amplified using a specific primer with a barcode. The PCR reactions were performed using TransStart FastPfu DNA Polymerase (TransGen, Beijing, China). Fluorescence quantification of PCR amplification recovery products was performed using the Quant-iT PicoGreen dsDNA Assay Kit. Sequencing libraries were generated with TruSeq Nano DNA LT Library Prep Kit for illumina, and sequenced by the MiSeq platform in the double-ended sequencing mode. The statistical tests were calculated using the Quantitative Insights into Microbial Ecology (QIIME) pipeline.

1. **Supported Tables**

| **Genes** | | **Forward primer** | **Reverse primer** |
| --- | --- | --- | --- |
| Nrf2 | AGTGGATCTGTCAGCTACTC | | GGCAAGCGACTGAAATGTAG |
| HO-1 | GCTTTGAAGAACCACCCAG | | GTCGGCCTGGCCTCTG |
| β-actin | | CACTTTCTACAATGAGCTGCG | CAGAGGCATACAGGGACAAC |

**Table S1. Primers used in the RT-PCR analysis.**

| **Weight loss (%)** | **Stool consistency** | **Occult/gross blood** | **Score** |
| --- | --- | --- | --- |
| None | Normal | Negative | 0 |
| 1-5 | / | + | 1 |
| 5-10 | Loose stools | ++ | 2 |
| 10-15 | / | +++ | 3 |
| ˃15 | Diarrhea | Gross bleeding | 4 |

**Table S2. Parameters, grades, and scores of DAI**. Five grades of weight loss (0, no weight loss or weight gain; 1, 1%-5% loss; 2, 5%-10% loss; 3, 10%-15% loss; 4, ˃15%); three grades of stool consistency (0, normal; 2, loose stool; 4, diarrhea); five grades of occult blood (0, negative; 1, +; 2, ++; 3, +++; 4, gross bleeding).

1. **Supported Figures**

**
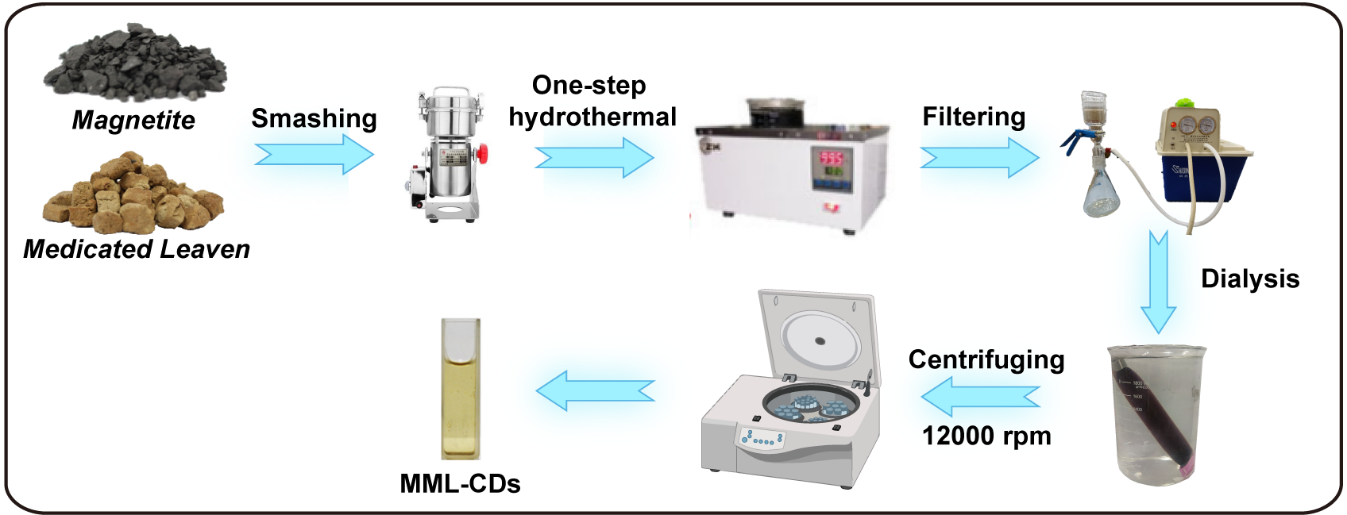
**

**Fig. S1**. The detail processing flowchart of MML-CDs.

**
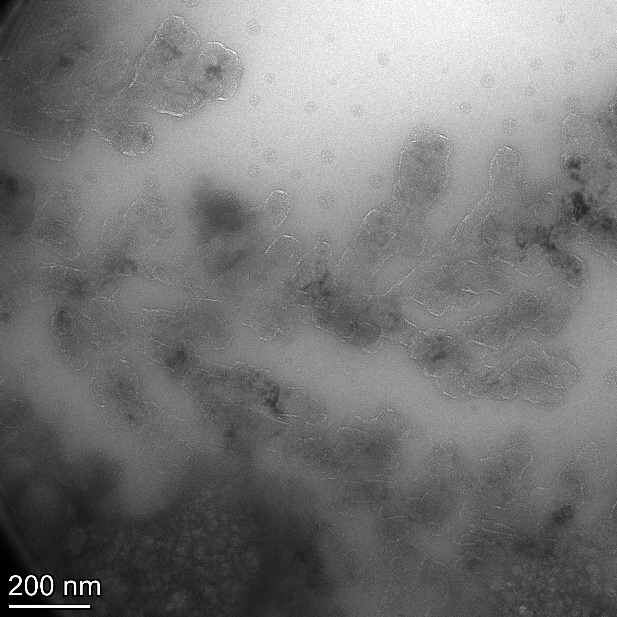
**

**Fig. S2**. The morphological characterization of pure ML solution.


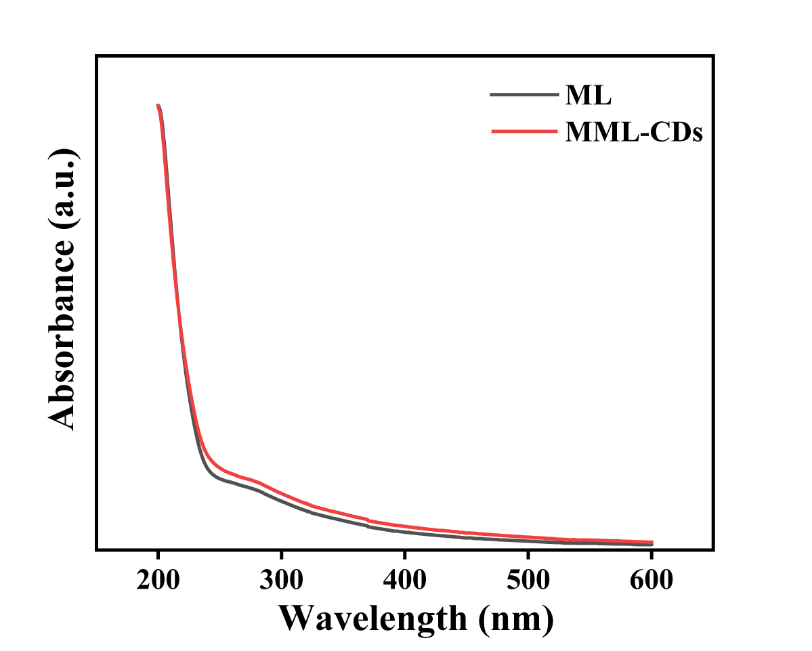


**Fig. S3.** The UV-vis spectra of MML-CDs from 200 to 600 nm.

**
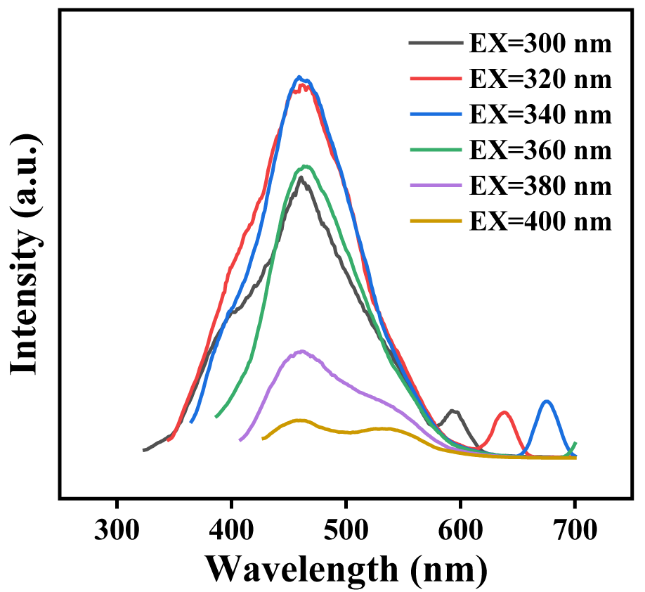
**

**Fig. S4.** The FL spectra of MML-CDs in different wavelength range 300 to 400 nm.


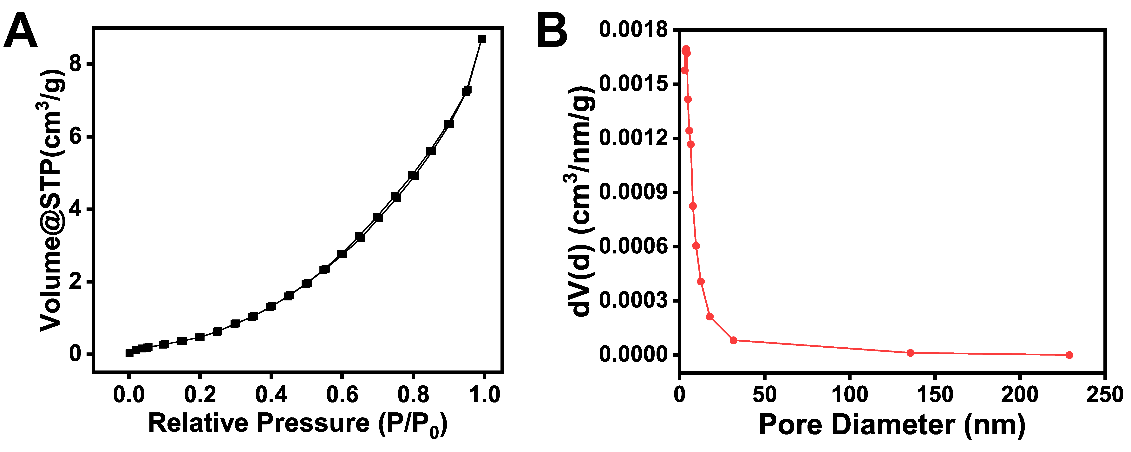


**Fig. S5**. BET surface area analysis. (A) BET plot of the surface area of MML-CDs with the N2 isotherm (B) Pore size distribution of MML-CDs.

**
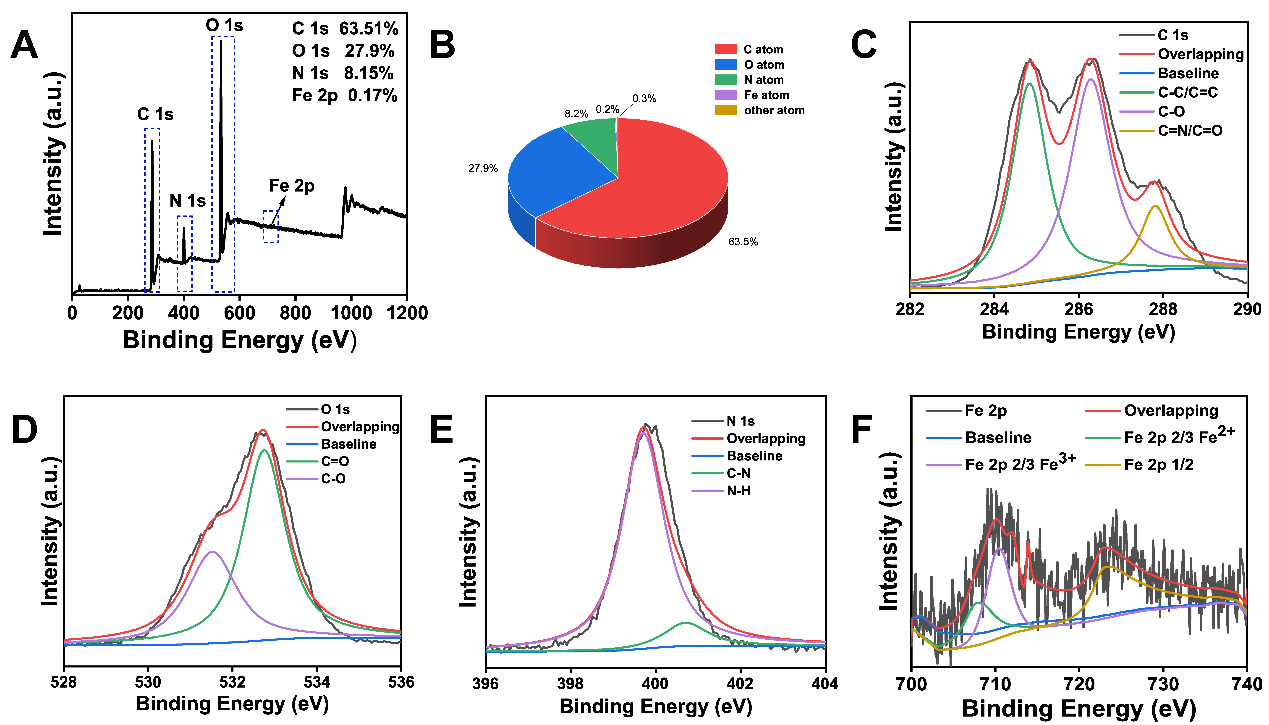
**

**Fig. S6**. The XPS spectra of MML-CDs. (A) C 1s XPS spectrum (B) O 1s spectrum (C) N 1s spectrum.


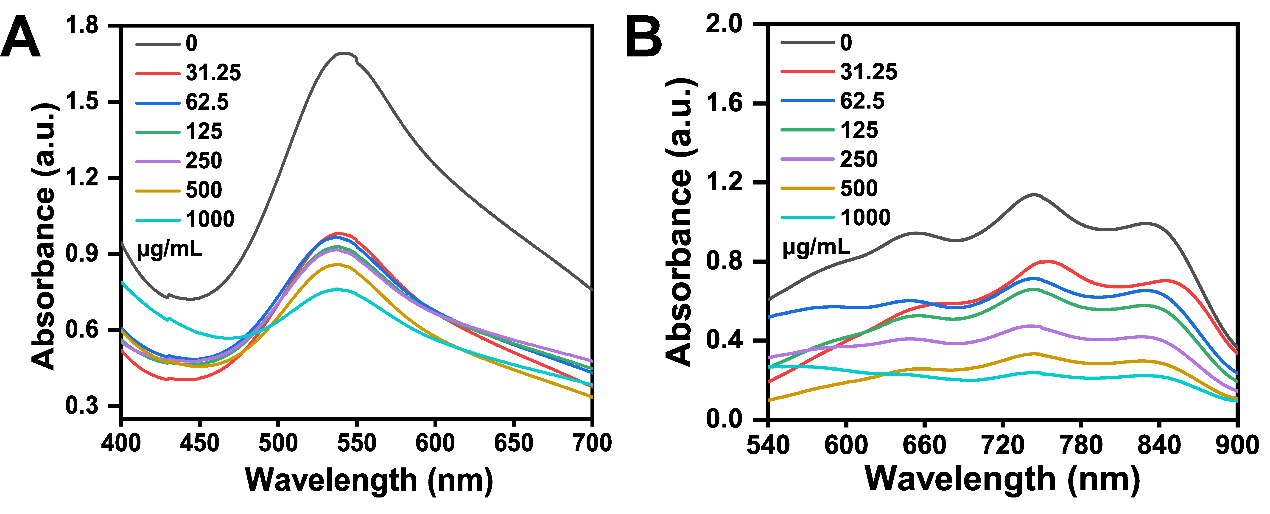


**Fig. S7** UV-vis absorbance spectra of DPPH· and ABTS+·. (A) UV-vis absorbance spectra of DPPH· radicals after incubation with different concentrations of MML-CDs. (B) UV-vis absorbance spectra of ABTS+· radicals after incubation with different concentrations of MML-CDs.


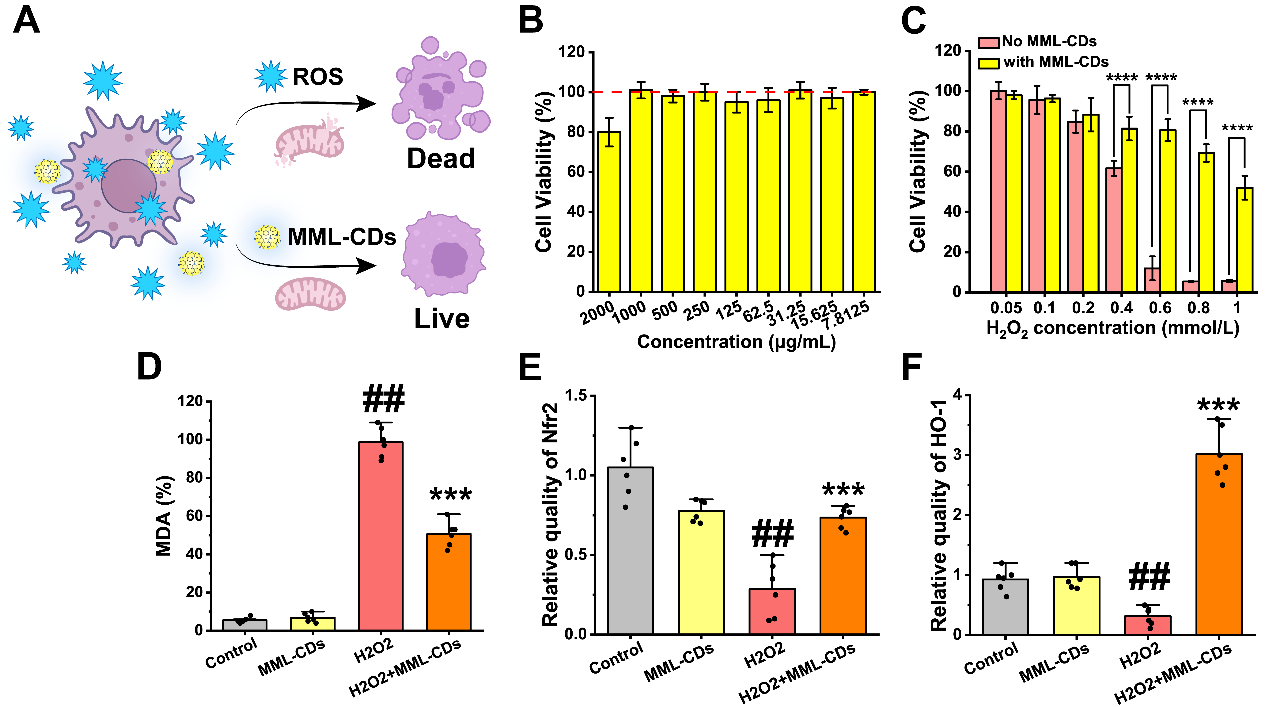


**Fig. S8**. Cellular anti-oxidant ability of MML-CDs on Caco-2 cell. (A) Schematic illustration of intracellular ROS scavenging and protective abilities. (B) Relative viabilities of Caco-2 cells after incubation with various concentrations of MML-CDs for 24 h. (C) Relative viabilities of Caco-2 with or without MML-CDs under various concentrations of H_2_O_2_. (D) MDA expression of Caco-2 cells received different treatment. (E, F) The mRNA expressions of Nrf2 and HO-1 on Caco-2 cells (*n*=3). Data are presented as mean ± SD and analyzed with one-way ANOVA followed with Tukey post hoc test (*n*=6 for biologically independent samples for (B and C), *n*=3 for biological independent samples for (D, E, and F). ^##^*P* < 0.01, ^*^*P* < 0.05, ^**^*P* < 0.01, ^***^*P* < 0.001, ^****^*P* < 0.0001.

**
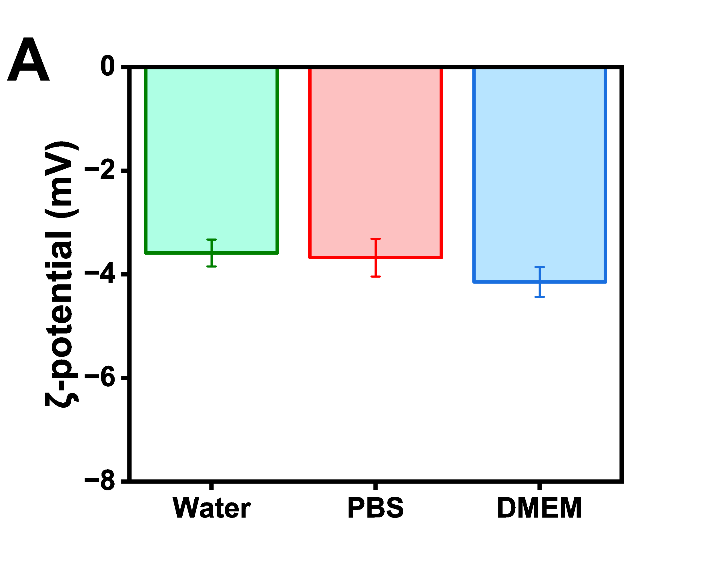
**

**Fig. S9** ζ-potential of MML-CDs in water, PBS and DMEM


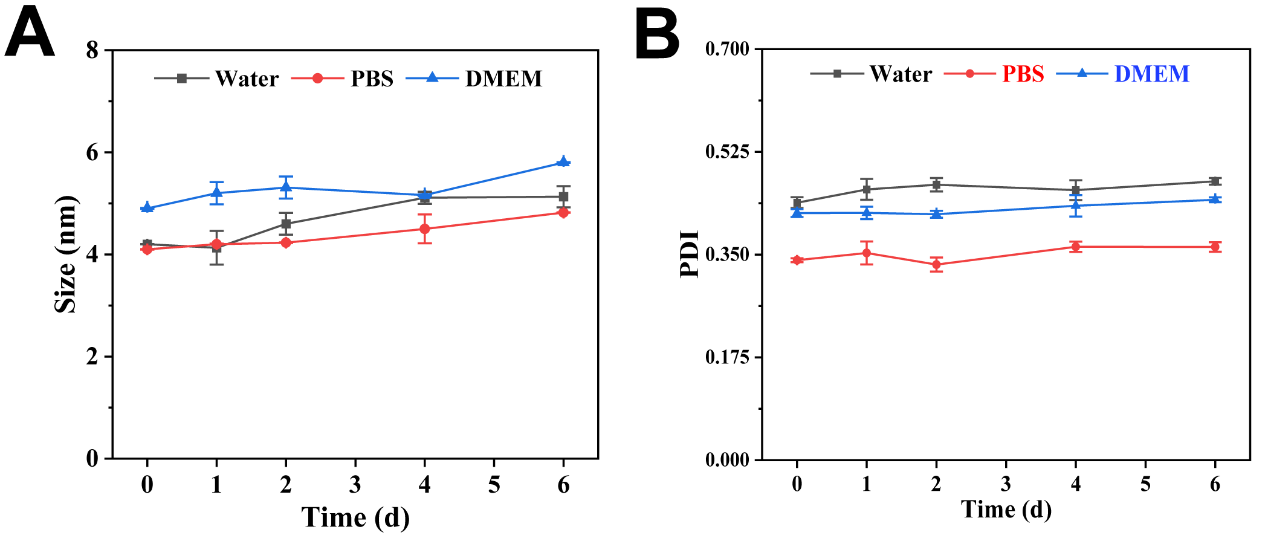


**Fig. S10** Stability of MML-CDs in different fluids within 6 days. (A) Hydrodynamic diameters (B) PDI

**
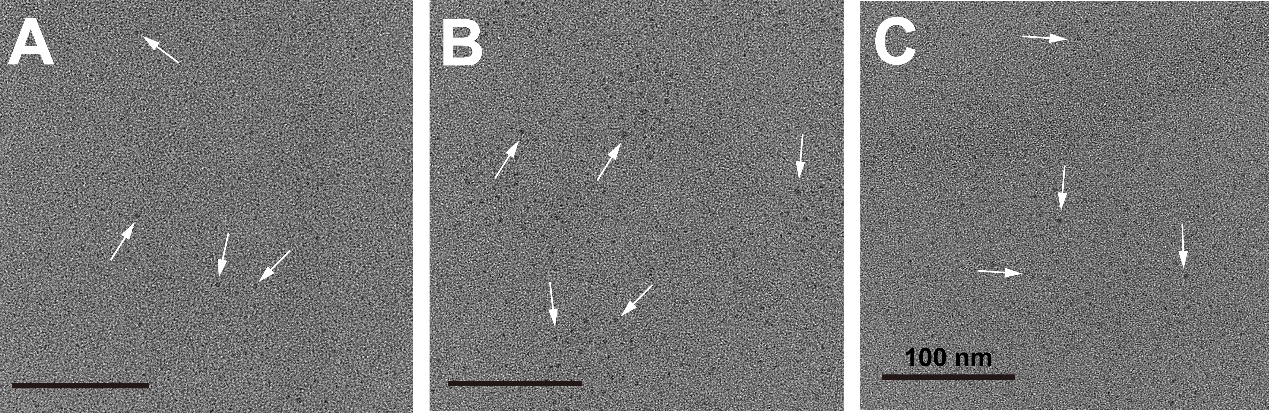
**

**Fig. S11** TEM images of MML-CDs in different digestive tract (white arrow: TEM appearance of carbon dots)

**
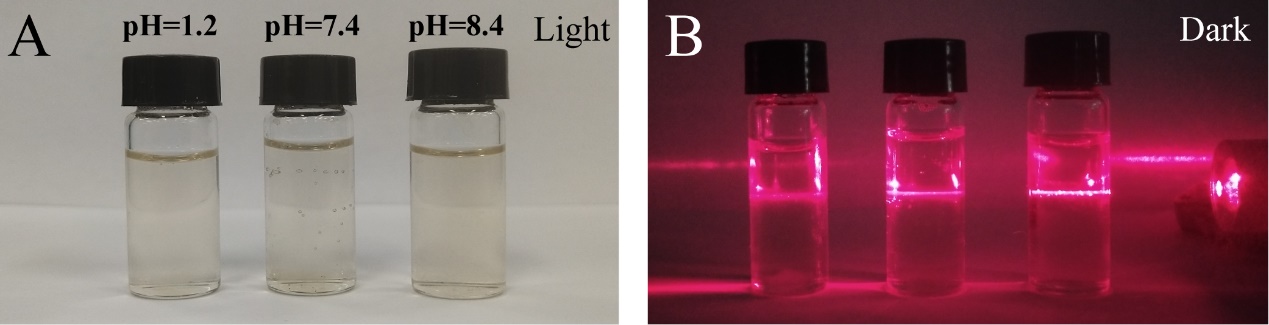
**

**Fig. S12.** (A) Solution appearance and (B) Tyndall effect of MML-CDs in GI fluids.

**
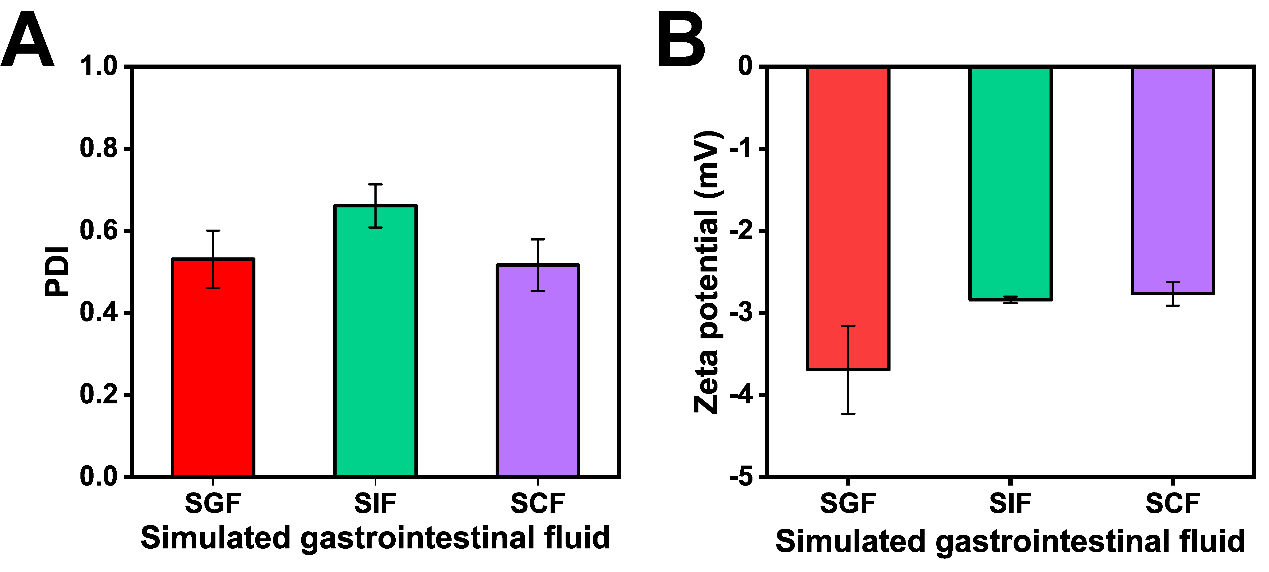
**

**Fig.S13** (A) Polymer dispersity index (PDI) of MML-CDs in different simulated gastrointestinal fluids (n=3). (B) ζ-potential of MML-CDs in different simulated gastrointestinal fluids (*n*=3).


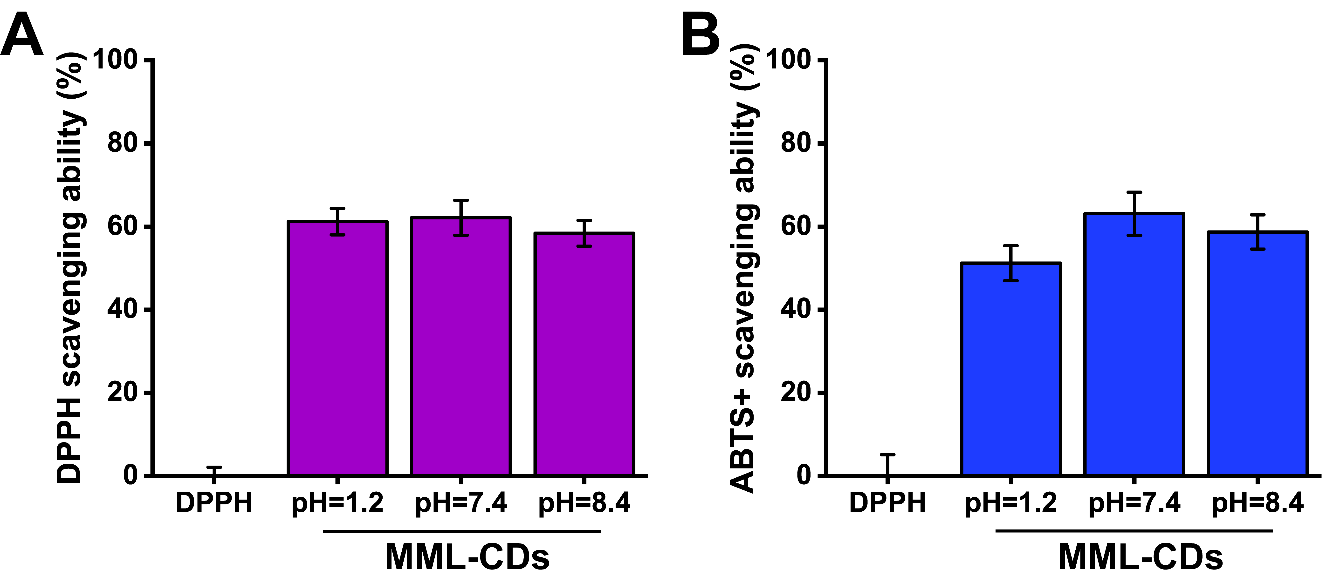


**Fig. S14** (A) DPPH·and (B) ABTS+·scavenging ability of various concentrations of MML-CDs in GI fluids.


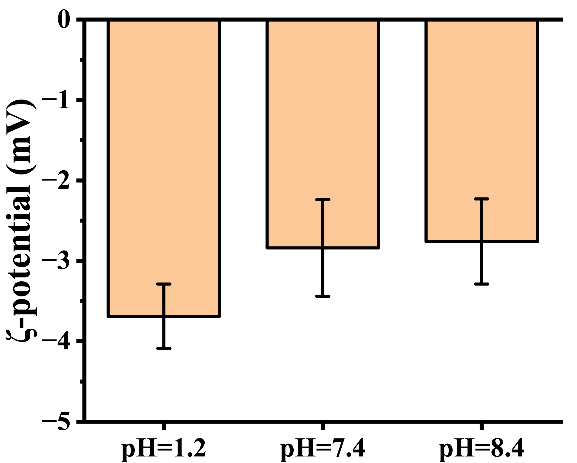


**Fig. S15** Zeta potential (ZP) within 20 days in GI fluids.


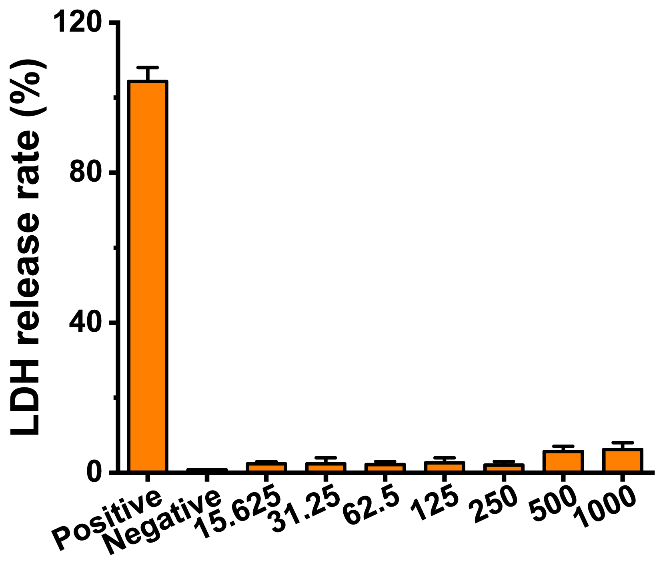


**Fig. S16** LDH release rates of Caco-2 after being co-incubated with different concentrations of MML-CDs.

**
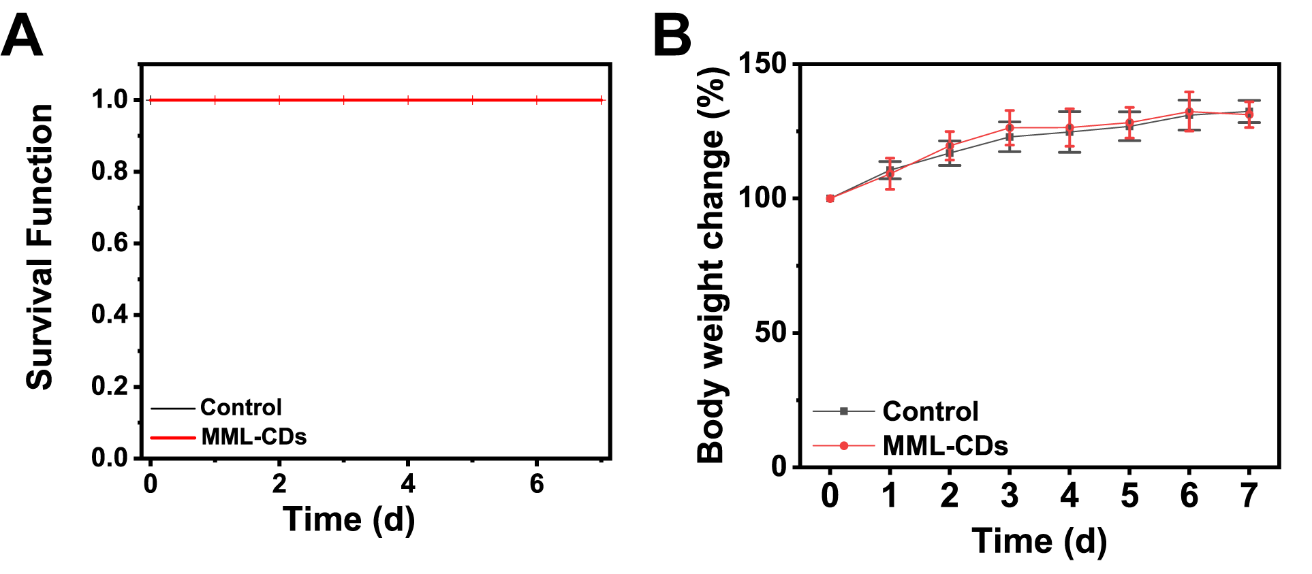
**

**Fig. S17** The survival curves (A) and (B) delta body weight change.(n=8)


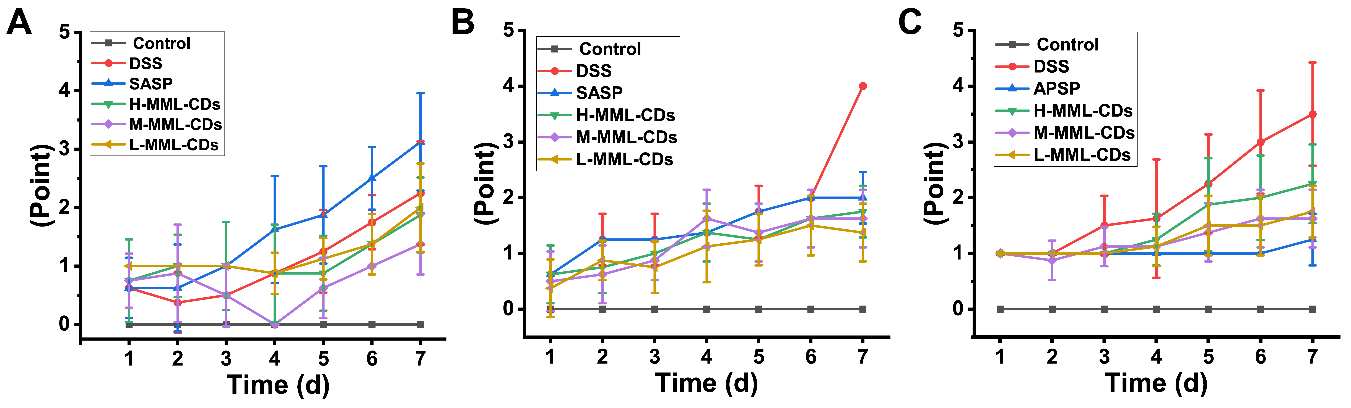


**Fig. S18** Therapeutic effects of DSS-induced UC in each group. DAI scoring of the DSS-induced UC. (A) Body weight change (B) Stool characteristics (C) Hematochezia


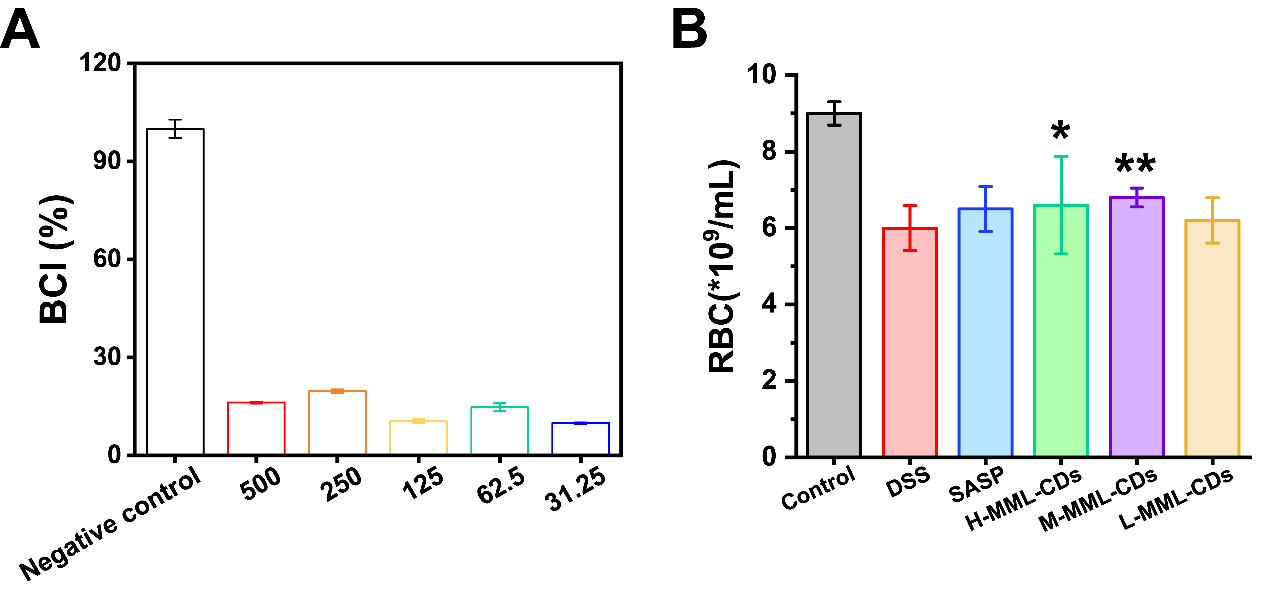


**Fig. S19** (A) BCI and (B) RBC amount.

**
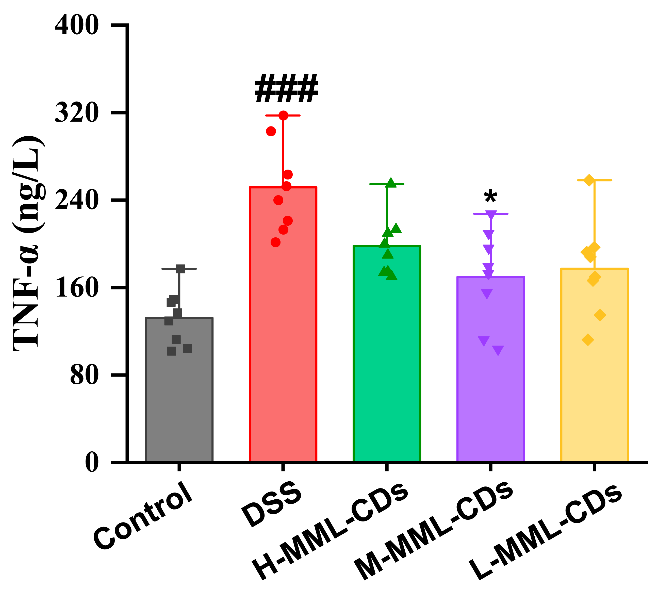
**

**Fig. S20** Inflammation cytokines expression levels of TNF-α in colon tissue. (*n*=8) ^###^*P* < 0.01, ^*^*P* < 0.05.

**
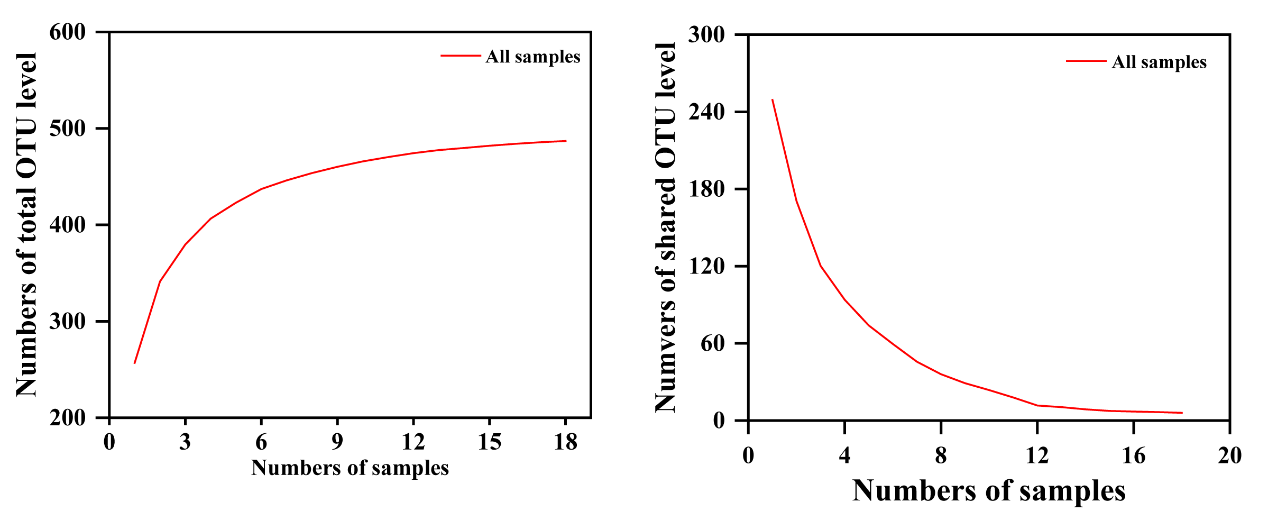
**

**Fig. S21** α diversity dilution curve.


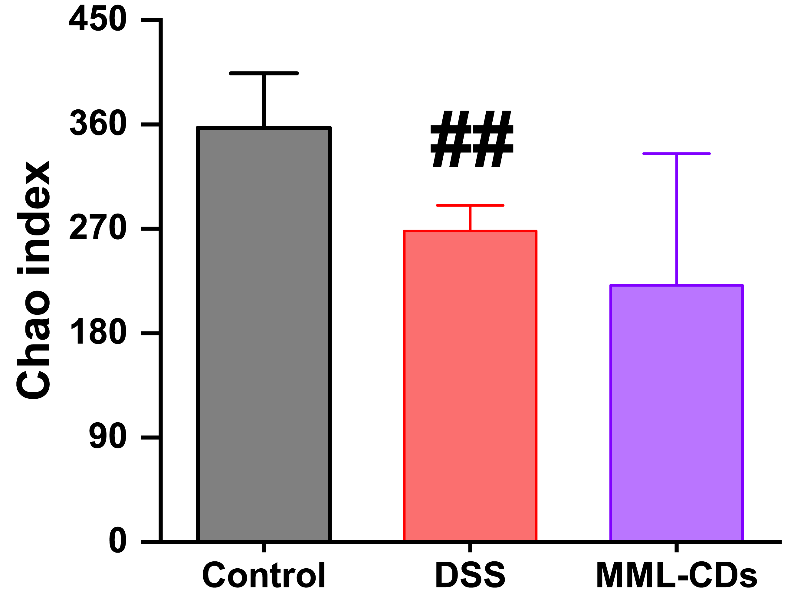


**Fig. S22** Chao index in different group (n=4).


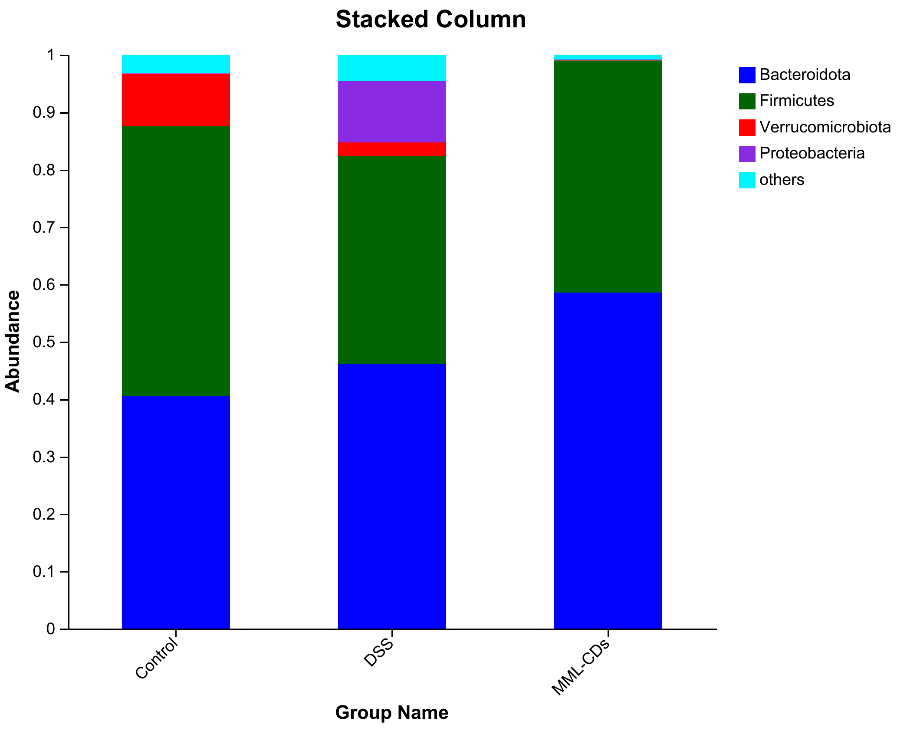


**Fig. S23** Relative abundance of species at phylum in each group (n=4).


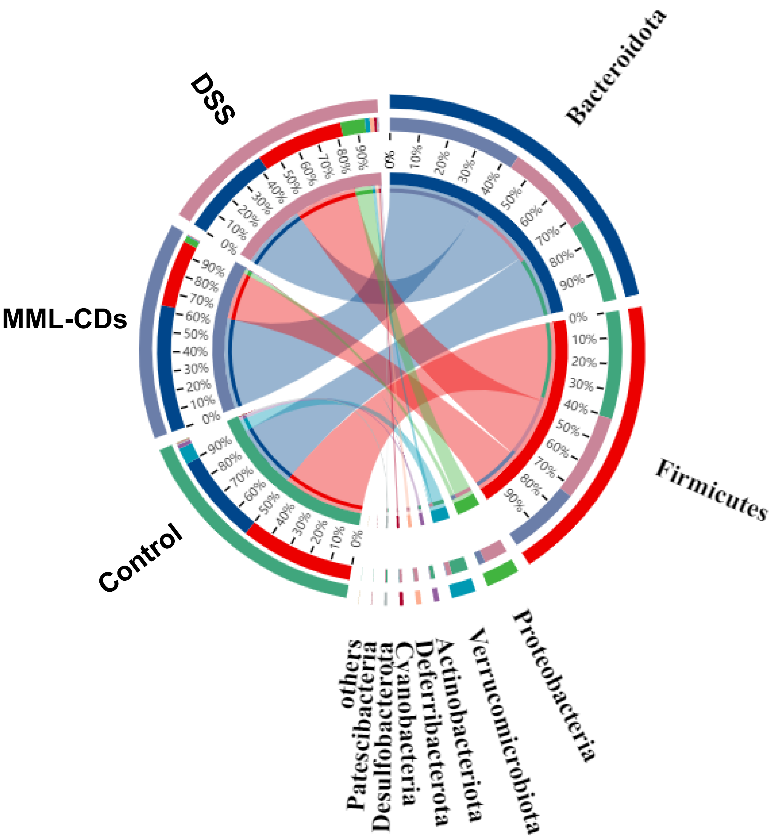


**Fig. S24** Circus plot of different group (n=4).


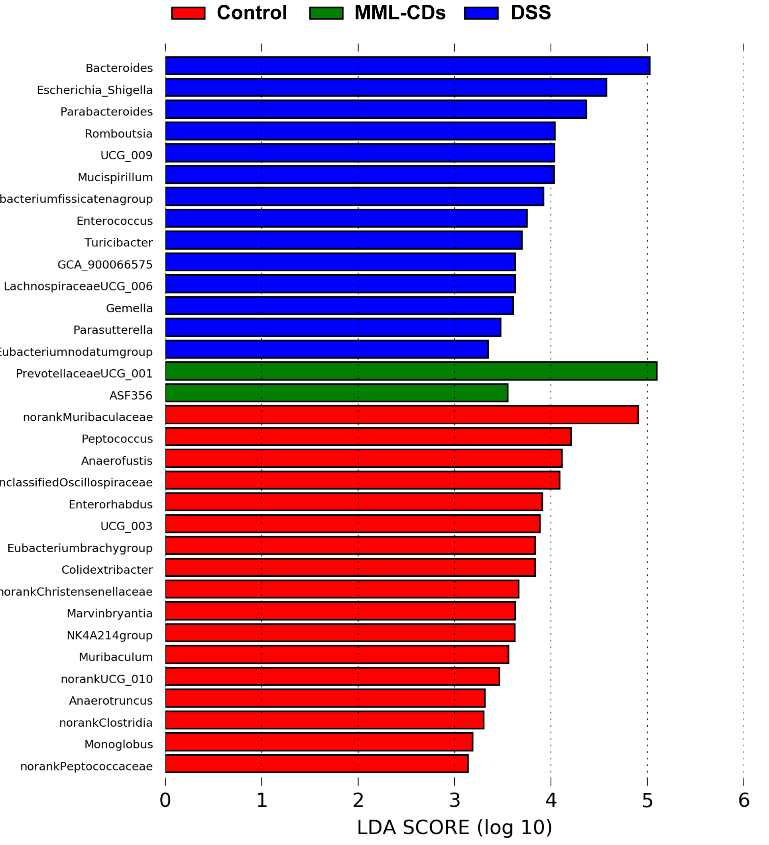


**Fig. S25** LDA effect size (LEFSe) analysis of the difference in the microbial community abundance of the intestinal flora. (n=4)


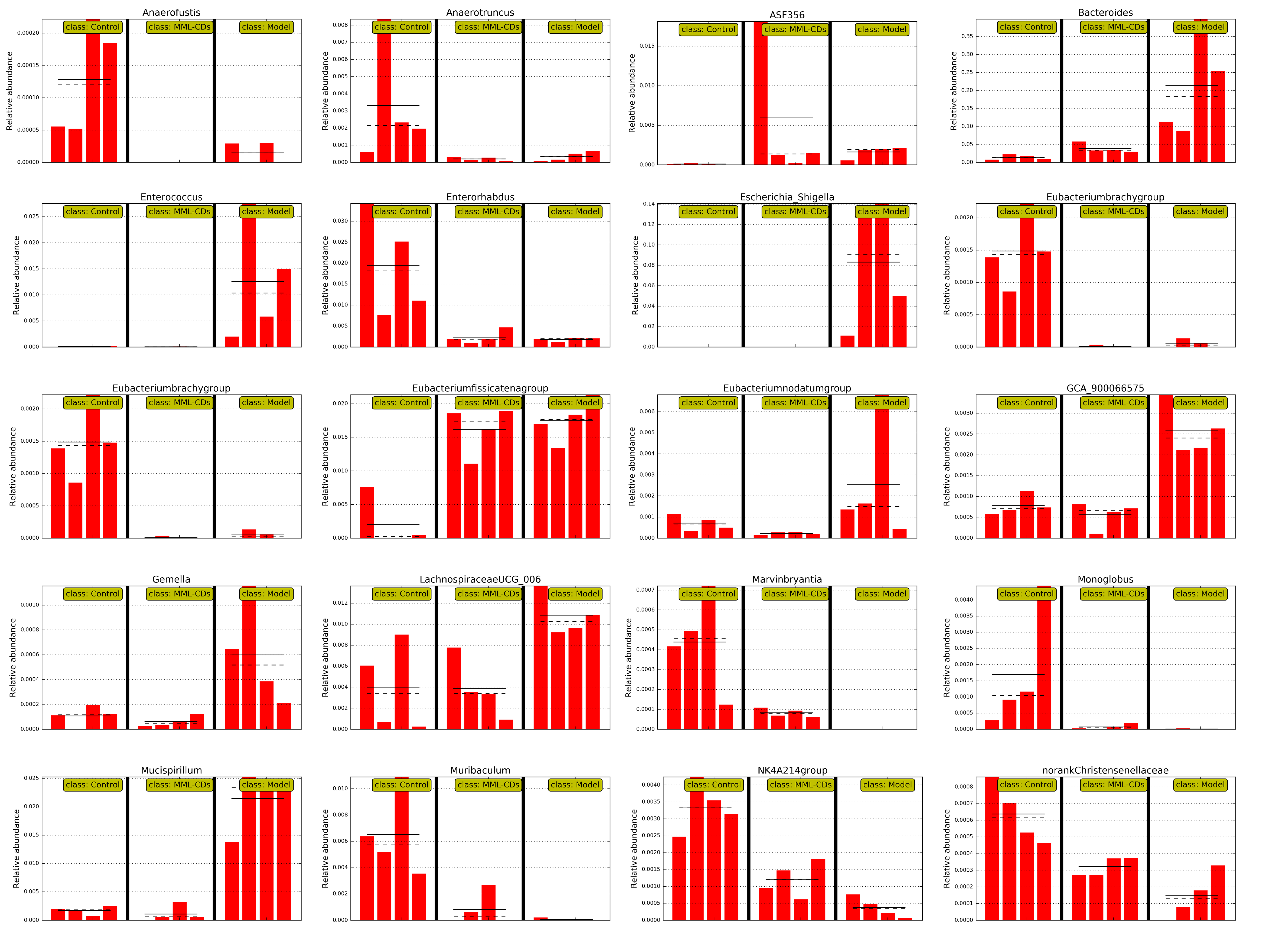


**Fig. S26** Characteristic histograms in the microbial community abundance of the intestinal flora (n=4).

**
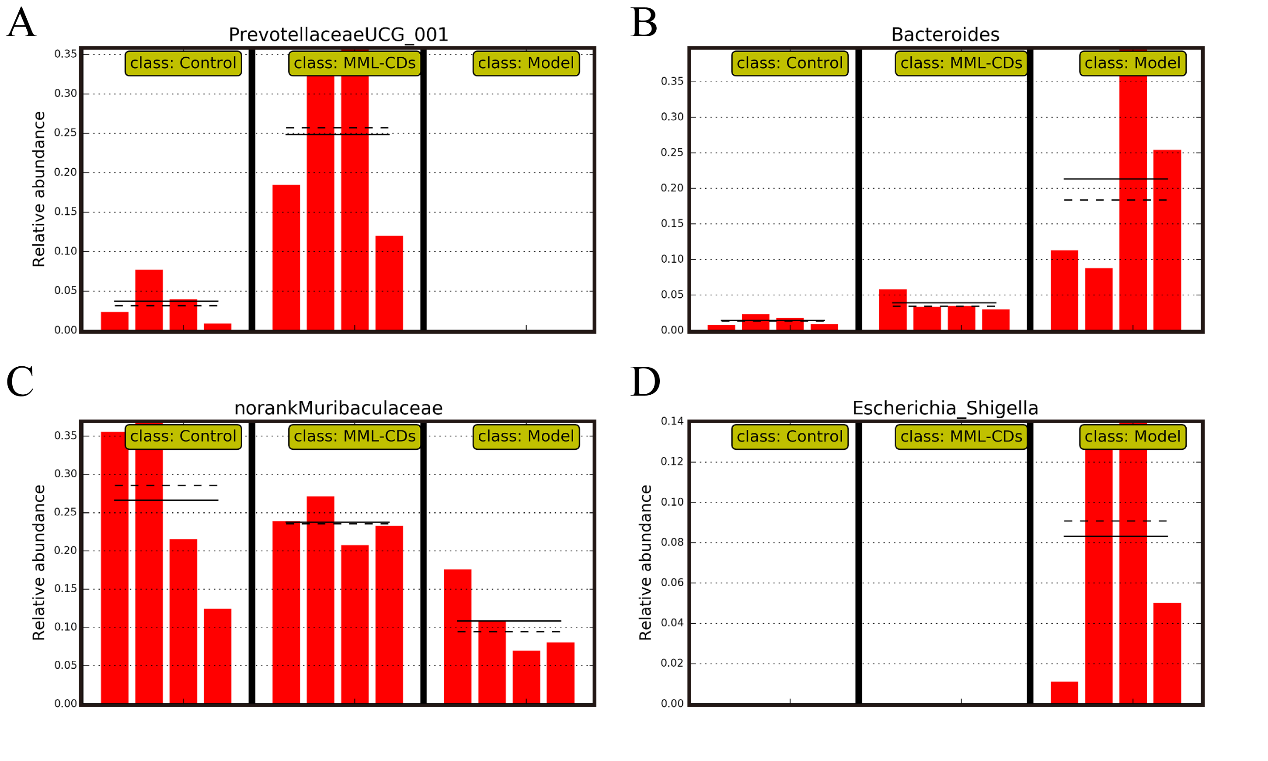
**

**Fig. S27** Characteristic histograms of *Prevotellaceae UCG 001*, *Bacterodes, norank*, *Muribaculaceae* and *Escherichia Shigella*.


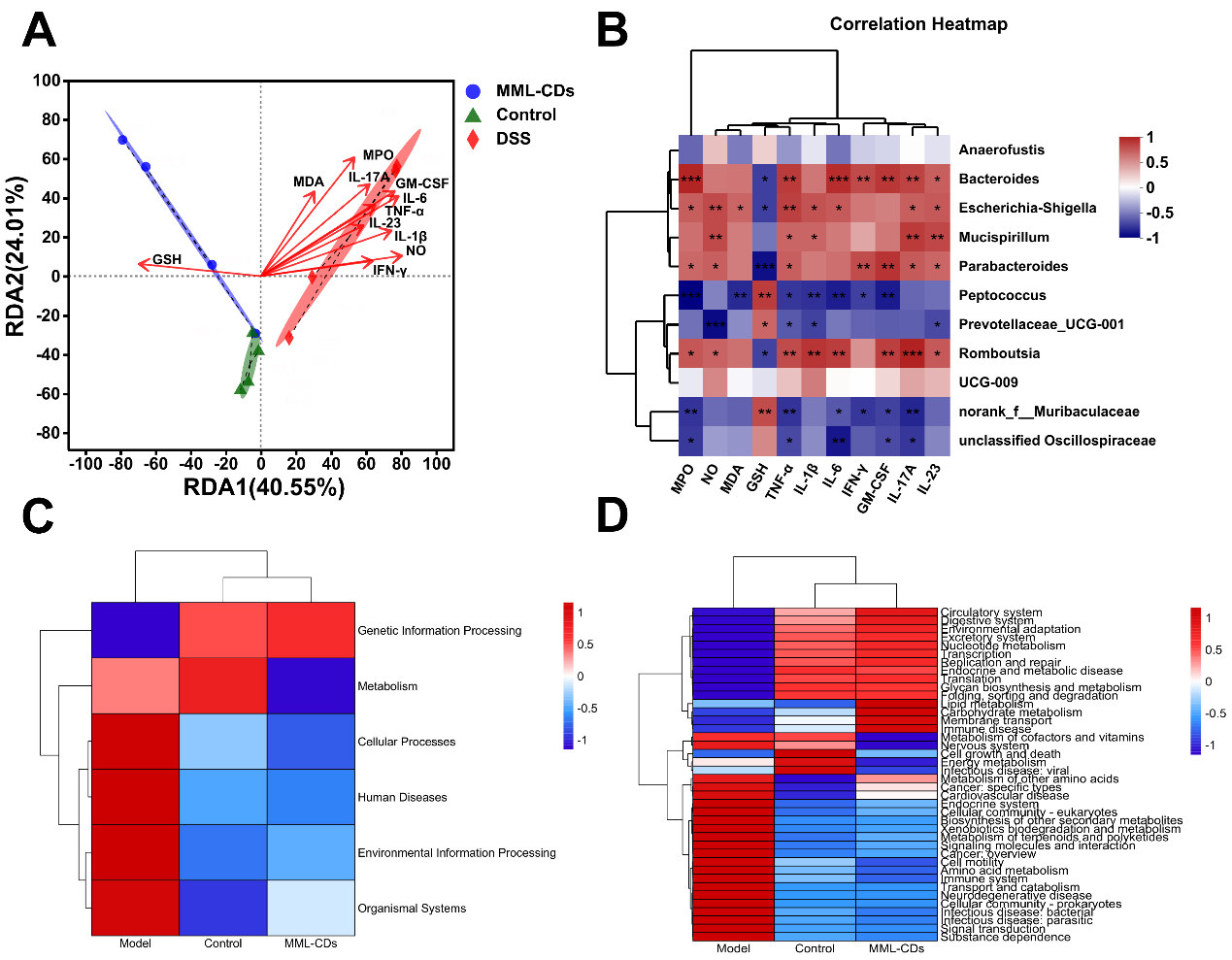


**Fig. S28** Correlation analysis and predicting functional analysis. (A) RDA analysis; (B) spearman correlation heatmap between intestinal flora and inflammation factors; (C, D) predicting functional heatmap in KEGG pathway level 1 and 2.
